# ERAD Component MoHrd3 Facilitates Pathogenicity and Establishes a Direct Regulation on Autophagy in *Magnaporthe Oryzae*

**DOI:** 10.1002/advs.202520627

**Published:** 2026-02-23

**Authors:** Huiqing Xia, Yunna Zheng, Nan Yang, Jing Chen, Xi Wu, Ximing Zheng, Danrui Cui, Ruijin Wang, Xunli Lu, Dongli Wang, You‐liang Peng, Qian Chen

**Affiliations:** ^1^ State Key Laboratory of Agricultural and Forestry Biosecurity, MARA Key Lab of Pest Monitoring and Green Management, College of Plant Protection China Agricultural University Beijing China

**Keywords:** autophagy, ERAD, magnaporthe oryzae, MoHrd3, MoAtg8, pathogenicity

## Abstract

Both ER‐associated degradation (ERAD) and autophagy play crucial roles in maintaining ER protein homeostasis. However, the regulatory relationship between ERAD and autophagy has remained unclear. Here, we report that MoHrd3 is an ERAD component that regulates autophagy in *Magnaporthe oryzae*, which causes devastating blast disease on rice and wheat. We found that *MoHrd3* is important for growth, conidiation, appressorium formation, and expansion of infection hyphae. Further studies showed that the autophagy level is reduced with an impaired fusion between the autophagosome and the vacuole in the *MoHrd3* deletion mutant. Interestingly, MoHrd3 directly interacts with MoAtg8, located on the autophagosome, and MoYpt7, situated on the vacuolar membrane. respectively. By serving as a mediator of the protein interaction between MoAtg8 and MoYpt7, MoHrd3 facilitates the fusion of these organelles. Further, we showed that the MoHrd3‐dependent fusion between the autophagosome and the vacuole is crucial for pathogenicity. In addition, MoHrd3 also works as an adaptor protein to promote the autophagic degradation of a GPCR protein MoPth11, which is required for appressorium formation and pathogenicity. Our discovery of MoHrd3's role in autophagy establishes a direct connection between ERAD and autophagy, revealing the intricate mechanisms governing protein quality control in this devastating plant pathogen.

## Graphical abstract

MoHrd3 plays a crucial role in regulating the pathogenicity of *Magnaporthe oryzae*. As an ER‐associated degradation component, MoHrd3 involves in ER stress‐triggered autophagy. It facilitates the fusion between the autophagosome and the vacuole via enhancing the interaction between MoAtg8 and MoYpt7. Additionally, MoHrd3 acts as an adaptor protein to facilitate the autophagic degradation of MoPth11.

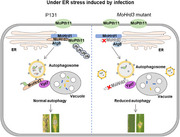

## 1 Introduction

Endoplasmic reticulum (ER)‐associated protein degradation (ERAD) is a conserved mechanism that alleviates ER stress by promoting the ER luminal‐localized or ER‐membrane anchored misfolded proteins into cytosolic proteasomal degradation [1]. This process involves E1, ER‐located ubiquitin‐conjugating enzymes (E2) and ubiquitin ligases (E3), which mediate the ubiquitination of cargoes recruited by adaptor proteins and lectin proteins, such as Hrd3 and Yos9 [2, 3]. After being ubiquitinated, the cargo proteins are transferred into the cytosolic proteasome with the help of the AAA‐type ATPase cell division control protein 48 (Cdc48)/p97 complex [1, 4]. ERAD is functionally conserved in eukaryotes and participates in signaling transduction and stress responses by regulating the stability of various cargo proteins from different signaling pathways [5].

Unfolded protein response (UPR) and autophagy are another two essential means to alleviate ER stress. For the functional consistency in facilitating cells recover from ER stress, there is precise regulation among ERAD, autophagy, and UPR in response to the dynamic stress levels of the cell. For example, a direct regulation between ERAD and UPR has been observed in mice, in which the ERAD‐related Hrd1‐Hrd3 complex (known as Sel1L in mammals) is responsible for the degradation of IRE1, a sensor of the UPR pathway [6]. This process occurs under normal conditions and is dampened during ER stress. Similarly, regulation within ERAD components is also seen in both plants and mammals, terming as ERAD tuning. In mammals, both gp78 and Hrd1 are orthologs of the yeast ERAD E3 ligase Hrd1p. In the ERAD tuning occurred in mammals, Hrd1 targets gp78 for proteasomal degradation, affecting the fate of gp78's substrate proteins differently [7]. In *Arabidopsis*, HRD1 binds to and degrades an ERAD related ubiquitin conjugating enzyme UBC32, while UBC32 reversely targets AtOS9, a component in HRD1 complex, for proteasome‐dependent degradation [8, 9]. These regulations occurred between ERAD‐ERAD or ERAD‐UPR ultimately reduce the ERAD or UPR activity under normal conditions. However, it is elusive whether there is a direct regulation between ERAD and autophagy, as seen between ERAD‐ERAD and ERAD‐UPR.

The ER membrane protein Hrd3, known as Hrd3p in yeast and Sel1L in mammals, is a conserved adaptor protein in the Hrd1 complex and serves as an “interaction scaffold” for substrate recognition in Hrd1‐dependent degradation [10, 11]. Despite its role in protein degradation, Hrd3 is crucial for the functional regulation of Hrd1. Current studies in yeast and mammals have demonstrated that Hrd3 helps maintain the stability of Hrd1 [10, 12]. Hrd1 itself and the protein USA1 are required for the degradation of Hrd1 in the absence of Hrd3 [12]. Moreover, the study of Hrd3 in human cells has revealed that it promotes the secretion of lipoprotein lipase (LPL), which is independently of its role in Hrd1‐mediated ERAD and ER homeostasis. In the absence of Hrd3, LPL forms aggregates that mainly degraded through autophagy. However, the degradation of LPL aggregates is impaired in Hrd3 knockout cells [13], suggesting that autophagy is suppressed in Hrd3‐deficient cells with an unknown mechanism.

According to the studies over the past decades, increasing evidences indicate that most autophagy cargoes are specifically selected by a type of receptor known as selective autophagy receptors [14]. These receptors bind to ATG8 through an ATG8‐interacting motif (AIM) consisting of four amino acids [W/F/Y]XX[L/V/I], and directly associate with cargoes or ubiquitin motifs attached to them [15]. Many selective receptors have been discovered until now, such as sequestosome‐1 (SQSTM1)/p62, neighbor of BRCA 1 (NBR1), optineurin (OPTN) in mammals, as well as NBR1 and RPN10 in plants [16]. The ERAD system also contributes to selective autophagy. In human cells, the ERAD‐related E3 ligase Hrd1 enhances the ubiquitination and autophagic degradation of SERPINA1^E342K^/ATZ (a misfolded protein that causes SERPINA1/AAT/α‐1antitrypsin deficiency), which depends on the selective autophagy receptor SQSTM1 [17]. However, it is still unknown whether ERAD components can directly function as selective autophagy receptors or adaptor proteins to mediate the autophagic degradation of target proteins.

*Magnaporthe oryzae* is an ascomycetous fungus that causes rice blast globally. ERAD components in *M. oryzae*, including MoHrd1, MoDer1, and MoCue1, have been characterized as important regulators in the growth and pathogenicity of *M. oryzae*, with largely limited mechanisms [18, 19]. In this study, we found that the ERAD component MoHrd3 is important for the growth, conidiation, and pathogenicity of *M. oryzae*. In the absence of MoHrd3, both appressorium formation and the growth of infection hypha are significantly reduced. We further showed that MoHrd3 participates in autophagy regulation. Notably, it directly interacts with MoAtg8 and the vacuolar membrane protein MoYpt7, promoting the fusion between the autophagosome and the vacuole by enhancing the interaction between MoAtg8 and MoYpt7. In addition, the MoHrd3‐dependent fusion between the autophagosome and the vacuole is crucial for pathogenicity in *M. oryzae*. Despite its role in ERAD, MoHrd3 also functions as an adaptor protein to mediate the autophagic degradation of MoPth11, following the ubiquitination mediated by the MoHrd1‐MoHrd3 complex. Collectively, our results indicate a direct interplay between ERAD and autophagy pathways, and highlight an essential role of MoHrd3 beyond its involvement in ERAD in *M. oryzae*.

## 2 Results

### 2.1 MoHrd3 is Essential for Growth, Conidiation, and Pathogenicity of *M. oryzae*

Hrd3 is an ERAD component conserved from yeast to higher eukaryotes and plays an important role in development and stress response. Interestingly, this key ERAD component also functions in ERAD‐independent manner in mammals [13]. Using the yeast Hrd3 as a query, we performed a pBLAST against the proteome of *M. oryzae*, showing that MGG_13508 in *M. oryzae* is the orthologue of Hrd3, named MoHrd3. To functionally characterize MoHrd3, we generated three deletion mutants of *MoHrd3* in the wild‐type strain P131 using a homologous recombination strategy and confirmed using Southern blot assays (Figure S1A,B). When cultured on oatmeal tomato agar (OTA) plates, the Δ*Mohrd3* mutant exhibited significantly reduced colony size compared to the wild‐type P131 strain (Figure S1C,D), indicating that MoHrd3 plays a crucial role in the growth of *M. oryzae*. Since the three mutants formed a similar‐size of colony, we selected one of them, named Δ*Mohrd3*, for further study.

**FIGURE 1:**
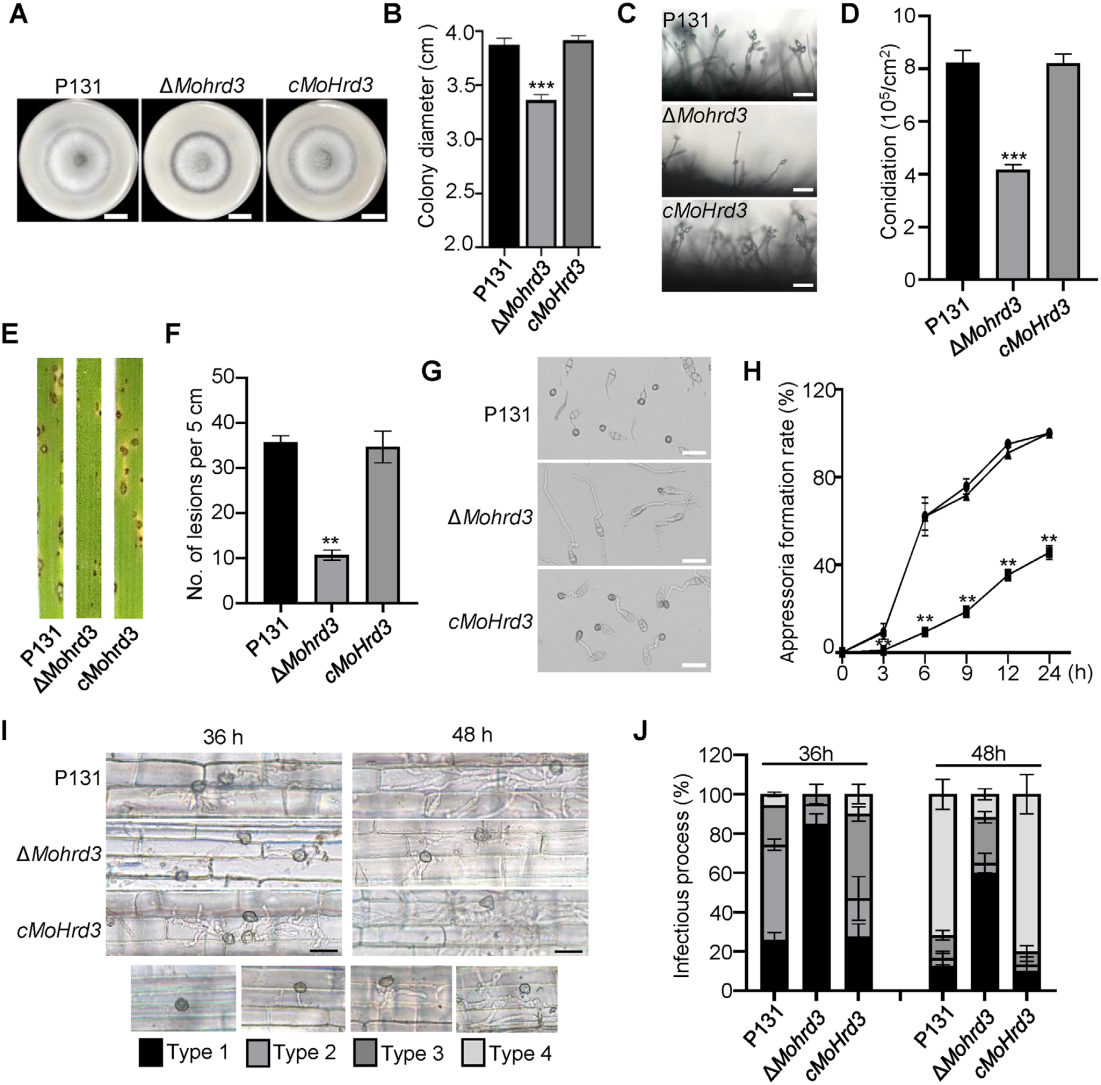
*MoHrd3* is required for growth, conidiation, and pathogenicity in *M.oryzae*. (A) The colony morphology of P131, Δ*Mohrd3*, and the complementation strain *cMoHrd3*. The strains were grown on OTA medium at 28°C for 5 days. Scale bar, 1 cm. (B) Mycelial growth diameter of P131, Δ*Mohrd3*, and *cMoHrd3* on OTA medium. Error bars represent SD, n = 3. The significant differences were evaluated by two‐tailed Student's *t*‐test, ^***^ *p* < 0.001. (C) Conidia production of P131, ∆*Mohrd3*, and *cMoHrd3* was observed under microscope. Scale bar: 20 µm. (D) Statistical analysis of conidia production of P131, Δ*Mohrd3*, and *cMoHrd3*. Error bars represent SD, n = 3. The significant differences were evaluated by two‐tailed Student's *t*‐test, ^***^ *p* < 0.001. (E) Rice seedlings were sprayed with conidial suspension (1.0 × 10^5^ spores/ml) of the indicated strains, and photographs were taken at 5 days post‐inoculation (dpi). (F) Statistical analysis of lesion numbers on rice leaves in (E). Error bars represent SD, n = 3. The significant differences were evaluated by two‐tailed Student's *t*‐test, ^**^ *p* < 0.01. (G) The appressorium formation of P131, Δ*Mohrd3*, and *cMoHrd3*. The indicated strains were incubated on coverslips for 6 h, and then the appressorium formation were observed. Scale bar, 20 µm. (H) The statistic data of appressorium formation in P131, Δ*Mohrd3*, and *cMoHrd3*. The indicated strains were incubated on coverslips for 0, 3, 6, 9, 12, and 24 h. Error bars represent SD, n = 300. The significant differences were evaluated by two‐tailed Student‘s *t*‐test, ^**^ *p* < 0.01. (I) Infection process of the indicated strains in rice cells was observed at 36 hpi and 48 hpi. The conidial suspension (1.0 × 10^5^ spores/ml) of the indicated strains was dropped on rice leaf sheath, and the infection process was observed at 36 hpi and 48 hpi, respectively. Scale bar, 20 µm. (J) Statistical analysis of infection hyphae in the rice cells infected by the indicated strains in (I). Four types of infection hyphae (type 1, only appressorium; type 2, appressorium with penetration peg; type 3, appressorium with invasive hypha > 1; type 4, invasive hypha was expanded to the next plant cell) were quantified. Error bars represent SD, n = 300.

When MoHrd3‐GFP driven by its native promoter was introduced into Δ*Mohrd3*, the transformants (named as *cMoHrd3*) completely restored colony growth to a level comparable to the wild‐type strain P131 (Figure 1A,B), with a similar colony thickness (Figure S2A). The complemented strains also recovered the conidiation capacity of Δ*Mohrd3* (Figure 1C,D). These results indicate that MoHrd3 is important for both the colony growth and conidiation of *M. oryzae*. Further, we performed infection assays to detect whether MoHrd3 affects the pathogenicity of *M. oryzae*. In the rice spray assay, more and bigger lesions formed on rice leaves inoculated with conidia of P131 and *cMoHrd3*, while only a few tiny lesions appeared on rice leaves inoculated with ∆*Mohrd3* (Figure 1E,F). In the wound assay, much smaller lesions were formed by ∆*Mohrd3* mutant compared with those by the P131 and *cMoHrd3* strains (Figure S2B,C). These assays verified that MoHrd3 is required for the pathogenicity of *M. oryzae*.

### 2.2 MoHrd3 is Required for Appressorium Maturation and Infection Hyphal Growth

To understand how MoHrd3 affects the pathogenicity of *M. oryzae*, we further compared the appressorium formation of P131, ∆*Mohrd3* mutant, and *cMoHrd3*. As shown in Figure 1G, more than 80% of P131 conidia formed melanized appressoria on hydrophobic coverslips at 6 h post‐inoculation (hpi), while the appressoria maturation rate of ∆*Mohrd3* mutant was less than 40%. The statistic data at 0, 3, 6, 9, 12, and 24 hpi further showed that appressorium formation in Δ*Mohrd3* is suppressed (Figure 1H). Therefore, MoHrd3 might contribute to pathogenicity by regulating appressorium formation of *M. oryzae*.

Then several indexes related to appressorium maturation and penetration, including glycogen and lipids utilization, septin ring formation and the appressorium collapse rate, were detected in P131 and ∆*Mohrd3* mutant. Using I_2_/KI and Nile red stain respectively, we found that both glycogen and lipids were completely utilized at 24 h in P131, whereas they were still be observed in the ∆*Mohrd3* mutant (Figure S3A–D), suggesting that *MoHrd3* is required for the efficient utilization of glycogen and lipids in *M. oryzae*. The septin ring formation, which involves the hetero‐oligomeric complexes of Sep3, Sep4, Sep5, and Sep6, is crucial to appressorial penetration [20]. Our results showed that both the Sep3‐GFP and Sep5‐GFP were localized to a ring structure in P131, while in the ∆*Mohrd3* mutant, they were dispersed in the appressoria (Figure S3E). Additionally, the appressoria collapse rate of ∆*Mohrd3* was significantly higher than that of P131 (Figure S3F), suggesting the turgor pressure in the ∆*Mohrd3* mutant is significantly reduced. Therefore, MoHrd3 contributes to multiple steps in appressoria maturation and penetration during the early stages of infection.

Besides its influence on appressoria, we also assessed the impact of MoHrd3 on the expansion of infectious hyphae in rice sheath. Statistical analysis showed that, at 36 hpi, approximately 70% of P131 or *cMoHrd3* in infected cells formed infectious hyphae with one or more branches, while over 80% of ∆*Mohrd3* only developed appressoria (Figure 1I,J). Therefore, the infection hyphal growth of *MoHrd3* mutant was significantly lower than that of the P131 and *cMoHrd3* strains, which is also consistent with the reduced lesion area observed in the wound‐drop assay with ∆*Mohrd3* mutant strains (Figure S2B,C). Together, these results demonstrated that MoHrd3 plays an important role in appressoria formation and infection hyphal growth.

### 2.3 MoHrd3 is a Conserved ERAD Component of MoHrd1 Complex in *M. oryzae*

**FIGURE 2:**
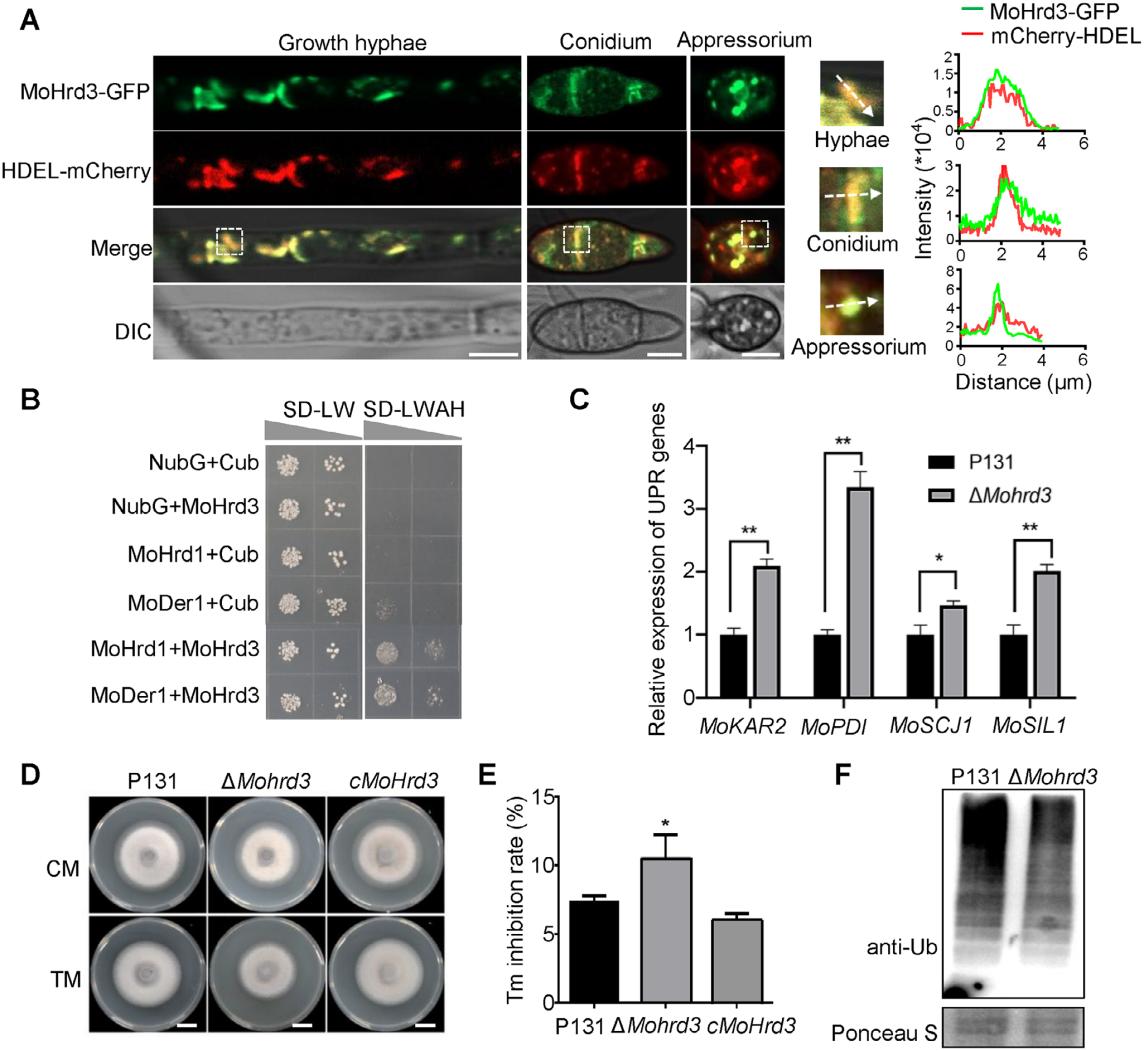
MoHrd3 is a conserved ERAD component of MoHrd1 complex in *M. oryzae*. (A) MoHrd3‐GFP was co‐localized with ER marker mCherry‐HDEL in growth hyphae, conidium, and appressorium. Scale bar, 5 µm. The white square with dotted lines indicated the areas used for linescan graph analysis. (B) MoHrd3 interacted with ERAD component MoHrd1 and MoDer1 by split‐ubiquitin yeast two‐hybrid system. NubG/Cub co‐transformation was used as negative controls. (C) The relative expression levels of UPR related genes in P131 and ∆*Mohrd3*. Error bars represent SD, the significant differences were evaluated by two‐tailed Student's *t*‐test, ^*^ *p* < 0.05; ^**^ *p* < 0.01. (D) Colony growth of P131, ∆*Mohrd3* and *cMoHrd3* on CM medium with or without 0.2 µg/ml TM. Scale bar: 1 cm. (E) The growth inhibition rate of strains in (d). Error bars represent SD, n = 3. The significant differences were evaluated by two‐tailed Student's *t*‐test, *p < 0.05. F) The ubiquitin level of total proteins was assayed in P131 and ∆*Mohrd3* mutant using anti‐ubiquitin antibody.

To investigate whether MoHrd3 associated with ERAD in *M. oryzae*, we generated MoHrd3‐GFP and ER marker mCherry‐HDEL constructs and co‐transformed them into ∆*Mohrd3* to examine the subcellular localization. As shown in Figure 2A, the GFP signal of MoHrd3‐GFP extensively overlapped with the ER marker mCherry‐HDEL in hyphae, conidia, and appressoria, confirming that MoHrd3 is an ER‐localized protein. Consistent with this localization and its proposed function, a split‐ubiquitin yeast two‐hybrid assay further demonstrated MoHrd3 direct interact with MoHrd1 and MoDer1 (Figure 2B), suggesting that they likely form an ERAD‐associated complex in *M. oryzae*.

Since functional deficiency of ERAD could invoke ER stress, leading to up‐regulation of unfolded protein response (UPR) genes, we detected the expression of UPR marker genes, including *MoKAR2* (MGG_02503), *MoPDI* (MGG_05753), *MoSCJ1* (MGG_07502), and *MoSIL1* (MGG_10843), in P131 and ∆*Mohrd3*. As shown in Figure 2C, all the UPR response genes were significantly induced in ∆*Mohrd3*, compared with in P131. Consistently, ∆*Mohrd3* mutant was more sensitive to tunicamycin (TM), an ER stress inducer that triggers ER stress by inhibiting protein N‐glycosylation (Figure 2D,E). As an essential ERAD component, Hrd3 recognizes substrates and facilitates their ubiquitination and degradation through the Hrd1‐dependent pathway [21]. Therefore, we compared global ubiquitination levels in P131 and ∆*Mohrd3*. We observed a significant decrease in total ubiquitinated proteins in Δ*Mohrd3* (Figure 2F), supporting a conserved role for MoHrd3 in substrate recognition in *M. oryzae*. Together, these results demonstrated that MoHrd3 in *M. oryzae* is an ERAD component of the MoHrd1 complex.

### 2.4 Loss Function of MoHrd3 Leads to Reduced Autophagy in *M. oryzae*

**FIGURE 3:**
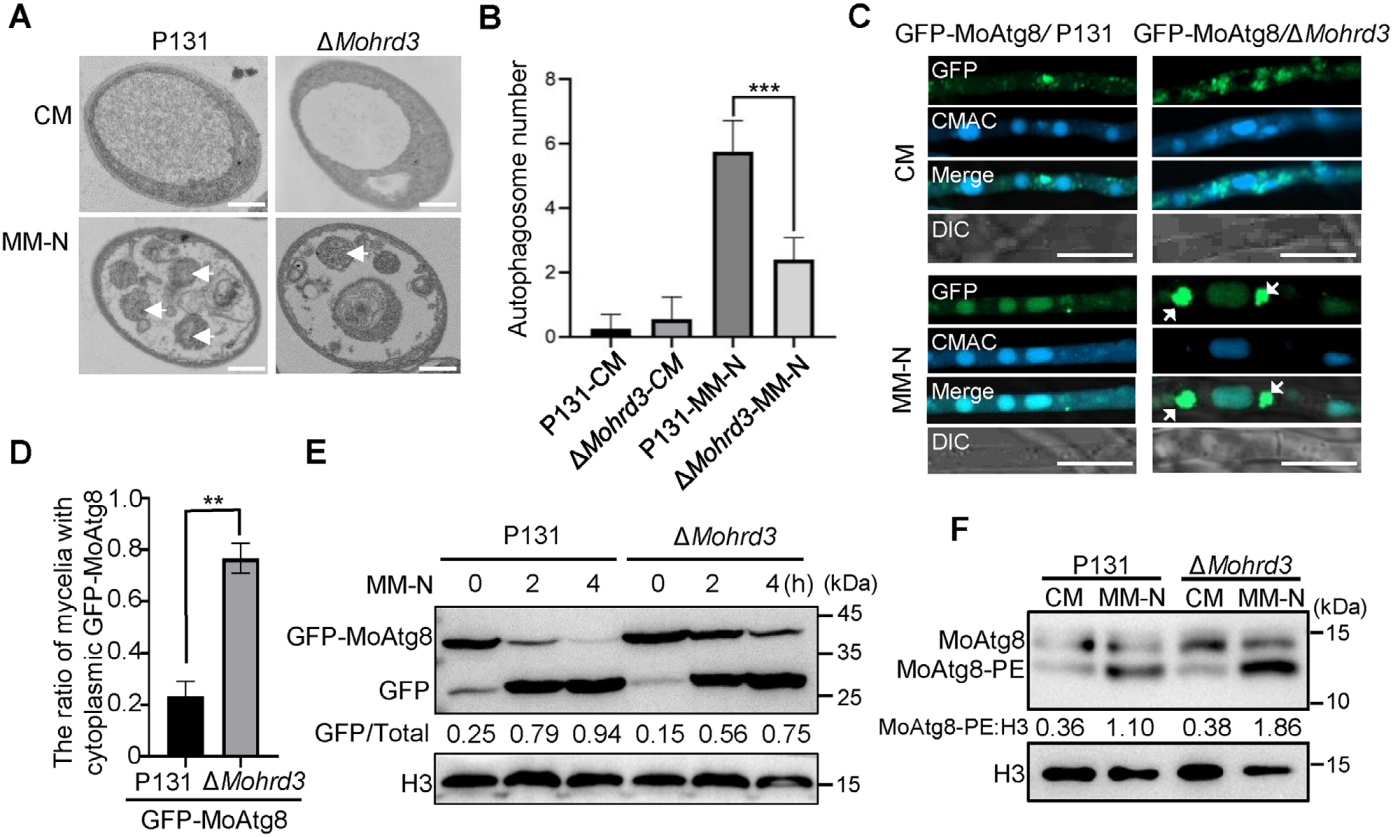
MoHrd3 positively regulates autophagy in *M. oryzae*. (A) The autophagosome in mycelia of P131 and ∆*Mohrd3* was observed under electron microscopy. P131 and ∆*Mohrd3* were grown in liquid CM for 36 h and then transferred to MM‐N medium for 5 h. The white arrows indicate autophagosomes in vacuole. Scale bar, 0.5 µm. (B) Statistical analysis of the autophagosome number of P131 and ∆*Mohrd3* in (A). Error bars represent SD, n = 10. Significant differences were evaluated by two‐tailed Student's *t*‐test, ^***^ *p* < 0.001. (C) The localization of GFP‐MoAtg8 in mycelia of P131 and ∆*Mohrd3*. GFP‐MoAtg8/P131 and GFP‐MoAtg8/∆*Mohrd3* were cultured in liquid CM for 36 h and then transferred to MM‐N medium for 5 h before microscope observation. Mycelia were stained with CMAC to indicate the vacuoles. The white arrow indicated GFP‐MoAtg8 puncta outside the vacuoles. Scale bar, 10 µm. (D) The statistic data of mycelia with cytoplasmic GFP‐MoAtg8 in (C). Error bars represent SD, n = 30. Significant differences were evaluated by two‐tailed Student's *t*‐test, ^**^ *p* < 0.01. (E) The breakdown of GFP‐MoAtg8 in P131 and ∆*Mohrd3*. The ratio of GFP to total protein (free GFP and GFP‐MoAtg8) was calculated using Image J software. (F) MoAtg8 lipidation and quantification analysis in P131 and *∆Mohrd3*. Strains were grown in liquid CM for 36 h and then transferred to MM‐N medium for 4 h.

To explore pathways regulated by MoHrd3, we performed LC‐MS/MS analysis on the MoHrd3‐GFP purified proteins. Interestingly, several sorting and vacuole membrane components closely related to autophagy were identified as candidate proteins interacting with MoHrd3 (Table S1). To test whether this ERAD component regulates autophagy, we compared the autophagy levels in P131 and ∆*Mohrd3*. We first determined the autophagosome number in mycelia of P131 and ∆*Mohrd3* cultured in complete medium (CM) and nitrogen‐limiting medium (MM‐N) by using transmission electron microscopy. Under CM conditions, there were few or no autophagosome formation in both P131 and ∆*Mohrd3* mycelial cells. However, after cultured in MM‐N for 5 h, a number of autophagosomes had accumulated in the vacuole of P131, whereas fewer autophagosomes were observed in the vacuole of the ∆*Mohrd3* mutant (Figure 3A,B), suggesting that MoHrd3 likely participates in regulating autophagy in *M. oryzae*.

The reduced autophagosome in vacuole of Δ*Mohrd3* mutant could result from either impaired fusion between the autophagosome and the vacuole or enhanced autophagic degradation. To distinguish these possibilities, we further compared the autophagosomes retained in the cytoplasm of P131 and Δ*Mohrd3* mutant. MoAtg8 is a marker widely used to detect autophagy levels. We introduced GFP‐MoAtg8 into P131 and ∆*Mohrd3*, respectively, and chose two transformants with similar gene expression level for further study (Figure S4A). When cultured in CM medium, few autophagosomes with weaker GFP‐MoAtg8 fluorescence were observed in the cytoplasm of P131, while more autophagosomes and stronger GFP‐MoAtg8 fluorescence were accumulated in the cytoplasm of ∆*Mohrd3* (Figure 3C). Consistent with the cellular fluorescence observation, the immunoblot assays showed that the protein level of GFP‐MoAtg8 is increased in ∆*Mohrd3* (Figure S4B). Following nutrient starvation induction in MM‐N, most GFP signal are observed in the vacuole of P131 (Figure 3C). However, some GFP signals are still detained in cytoplasm of ∆*Mohrd3* mutant (Figure 3C). The statistic data showed that the ratio of mycelia with cytoplasmic GFP‐MoAtg8 is significantly increased in ∆*Mohrd3* (Figure 3D), suggesting that the fusion between the autophagosome and the vacuole may be affected in the ∆*Mohrd3* mutant. Autophagic flux is also assessed by analyzing vacuolar delivery and subsequent breakdown of GFP‐MoAtg8. When grown in MM‐N medium, the ratio of free GFP to total protein (free GFP and GFP‐MoAtg8) was increased in P131, while a much lower ratio of free GFP to total protein was displayed in ∆*Mohrd3* mutant (Figure 3E). The turnover of endogenous Atg8/Atg8‐phosphatidylethanolamine (Atg8‐PE) is another monitor to indicate the level of autophagy. Therefore, we assessed the MoAtg8‐PE accumulation in P131 and the ∆*Mohrd3* mutant. The results in Figure 3F showed that the degradation of MoAtg8‐PE was inhibited in the ∆*Mohrd3* mutant compared with P131. Collectively, these results support that MoHrd3, a conserved ERAD component, plays an important role in autophagy regulation.

### 2.5 MoHrd3 Associates and Co‐Localizes with MoAtg8 in *M. oryzae*

**FIGURE 4:**
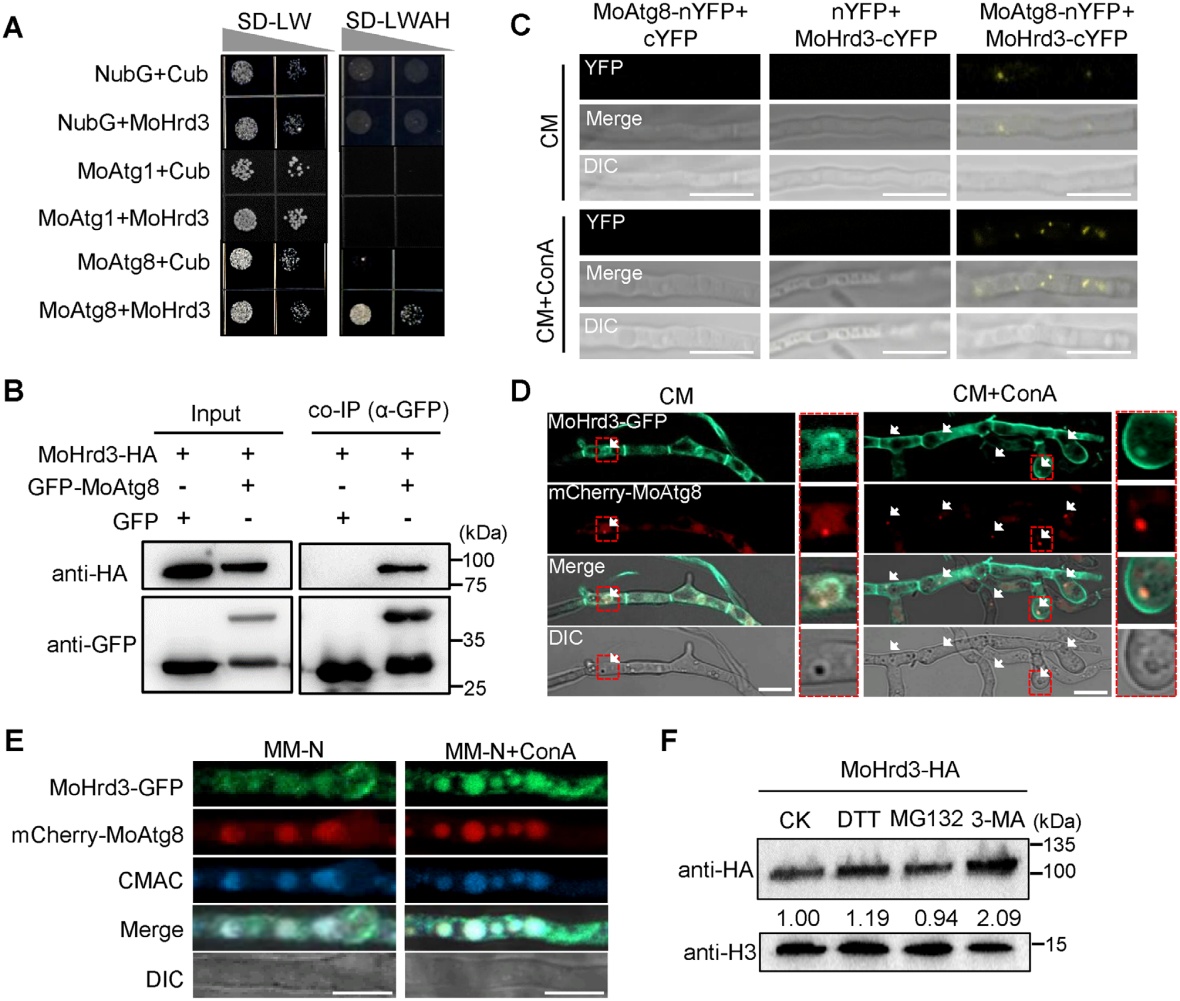
MoHrd3 interacts with MoAtg8 and is degraded through autophagy. (A) The split‐ubiquitin membrane yeast two‐hybrid system was used to test the interaction between MoHrd3 and MoAtg8. NubG/Cub co‐transformation was used as negative controls. (B) MoHrd3 interacted with MoAtg8 in a co‐IP assay. GFP‐MoAtg8 and MoHrd3‐HA plasmids were co‐transformed into P131. Total proteins were purified by GFP‐Nanoab‐Agarose beads and the products were detected using the indicated antibodies. (C) MoHrd3 associated with MoAtg8 in a BiFC assay. nYFP‐MoAtg8/MoHrd3‐cYFP and their related control pairs were co‐expressed in *M. oryzae*. Strains were cultured in liquid CM treatment with or without 1 µm ConA for 4 h before observation. Scale bar, 10 µm. (D) MoHrd3‐GFP co‐localized with mCherry‐MoAtg8 in growth hyphae. MoHrd3‐GFP and mCherry‐MoAtg8 were co‐transformed into P131. The generated strains were cultured in liquid CM medium with or without 1 µm ConA before observation. Scale bar, 10 µm. (E) MoHrd3‐GFP was co‐localized with mCherry‐MoAtg8 in hyphae. Strains were cultured in liquid MM‐N medium with or without 1 µm ConA. Mycelia were stained with CMAC to indicate the vacuoles. Scale bar, 10 µm. (F) MoHrd3 was degraded through autophagy pathway. MoHrd3‐HA/P131 were treated with DTT (10 mm), MG132 (100 µm), or 3‐MA (500 µm) for 10 h. The band intensity was calculated by Image J.

To investigate how MoHrd3 is involved in autophagy, we assessed the response of ATG genes to ER stress in P131 and ∆*Mohrd3* mutant strains. Among the ATG genes, *MoATG1*, *MoATG4*, *MoATG5*, *MoATG8*, and *MoATG9* could be induced by DTT (an ER stress inducer) treatment, and, in particular, *MoAtg1* and *MoAtg8* showed differential expression levels in P131 and the ∆*Mohrd3* mutant (Figure S5A). This suggested that MoAtg1 and MoAtg8 are involved in ER stress response in a MoHrd3‐regulated manner. Therefore, we examined whether MoHrd3 interacts with MoAtg1 and MoAtg8 by yeast two‐hybrid. As shown in Figure 4A, the results showed that MoHrd3 specifically interacts with MoAtg8, but not with MoAtg1. As a control, MoHrd1, which partners with MoHrd3 in the ERAD complex, showed no binding to MoAtg8 (Figure S5B), confirming the specificity of the MoHrd3‐MoAtg8 interaction. Their interaction is further determined using an in vivo co‐IP assay (Figure 4B). In addition, through a bimolecular fluorescence complementation (BiFC) assay, we found MoHrd3 interacts with MoAtg8 in the autophagosome (Figure 4C). When treatment with Concanamycin A (ConA), a V‐ATPase inhibitor that induces the accumulation of autophagosomes [22], their interaction signal was increased (Figure 4C).

The co‐localization of MoHrd3 and MoAtg8 in *M. oryzae* was also determined. To this end, we co‐transformed MoHrd3‐GFP and mCherry‐MoAtg8 in P131. In the CM medium, few autophagosome localization of MoHrd3‐GFP that merged with mCherry‐MoAtg8 could be observed (Figure 4D). Under ConA treatment, more MoHrd3‐GFP localized to autophagosomes in mycelia and appressoria (Figure 4D; Figure S5C). When the strains were treated under starvation conditions in the presence of ConA, both MoHrd3 and MoAtg8 are localized into vacuole, where the proteins were degraded (Figure 4E). This result promoted us to further examine whether MoHrd3 is also degraded through the autophagy pathway. MoHrd3‐HA transformant strains were treated with DTT, MG132 (a 26S proteasome inhibitor), and 3‐MA (an autophagy pathway inhibitor). The results showed that 3‐MA enhanced the protein accumulation of MoHrd3, while DTT and MG132 cause a slightly increase or no effect on MoHrd3 accumulation (Figure 4F). Therefore, MoHrd3 associates and co‐localizes with MoAtg8 in autophagosomes, and it could be degraded through autophagy.

### 2.6 MoHrd3 enhances the Interaction between MoYpt7 and MoAtg8 to Facilitate Autophagy

**FIGURE 5:**
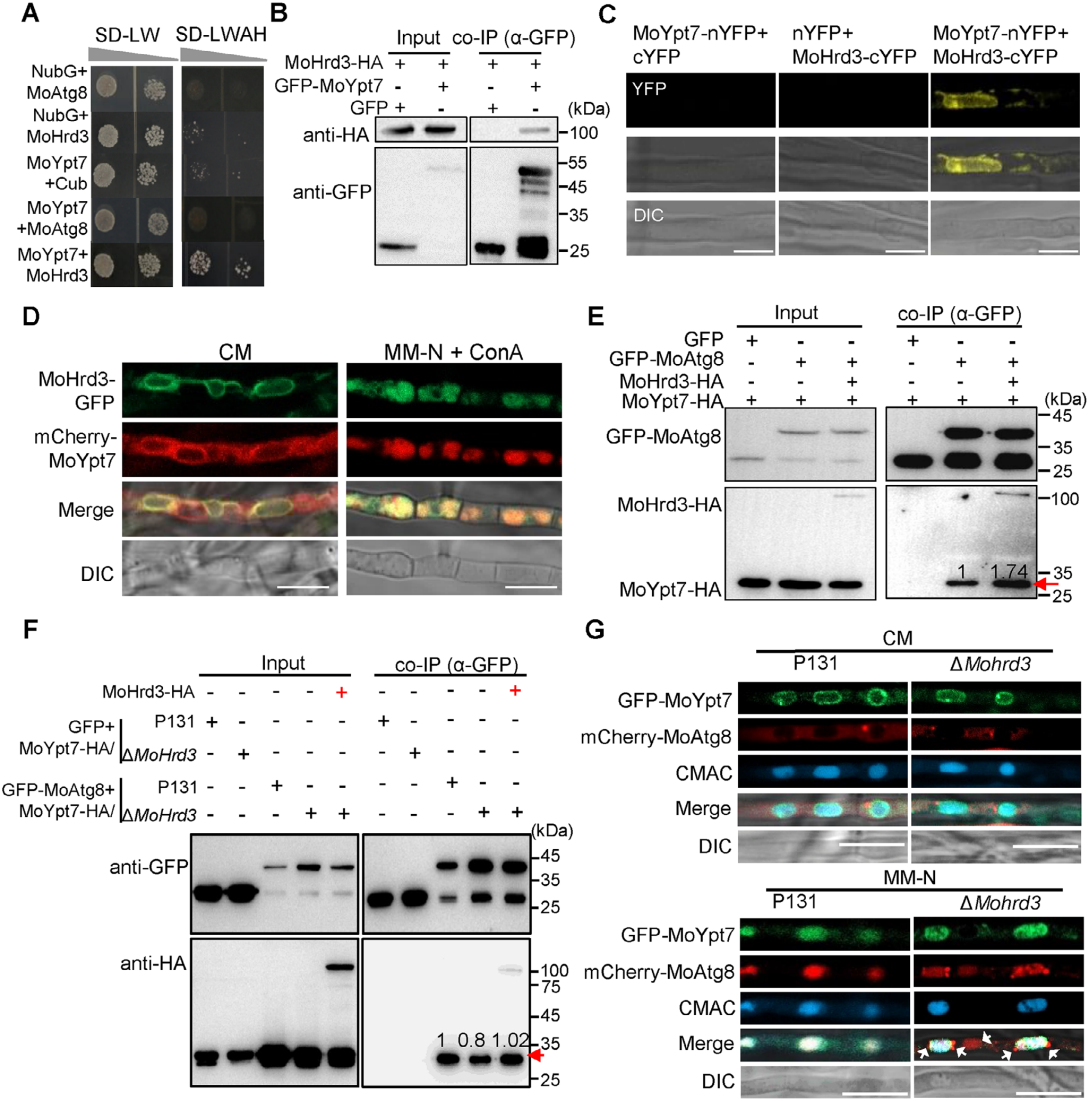
MoHrd3 interacts with MoYpt7 and enhances the association between MoYpt7 and MoAtg8. (A) The interaction between MoYpt7 and MoHrd3 was verified by yeast two‐hybrid. (B) MoHrd3 interacted with MoYpt7 in a co‐IP assay. MoHrd3‐HA and GFP‐MoYpt7 plasmids were transformed in P131 strain, respectively. The MoHrd3‐HA was detected following GFP‐MoYpt7 immunoprecipitation. (C) MoHrd3 associated with MoYpt7 by BiFC assays. nYFP‐MoYpt7/MoHrd3‐cYFP and their related control pairs were co‐expressed in *M. oryzae*. Strains were cultured in liquid CM for 36 h. Scale bar, 5 µM. (D) MoHrd3‐GFP co‐localized with mCherry‐MoYpt7 in growth hyphae. The strains co‐transformed with MoHrd3‐GFP and mCherry‐MoYpt7 were cultured in liquid CM medium with or without further treatment with MM‐N medium (with 1 µm ConA) for 5 h before observation. Scale bar, 10 µm. (E) MoHrd3 enhanced the interaction between MoYpt7 and MoAtg8 in a co‐IP assay. GFP‐MoAtg8/∆*Mohrd3*, MoHrd3‐HA/∆*Mohrd3* and MoYpt7‐HA/∆*Mohrd3* strains were used in this assay. The MoHrd3‐HA and MoYpt7‐HA were detected following GFP‐MoAtg8 immunoprecipitation. (F) Deletion of *MoHrd3* reduced the interaction between MoYpt7 and MoAtg8 in a co‐IP assay. GFP or GFP‐MoAtg8 plasmids were co‐transformed with MoYpt7‐HA plasmid in P131 or ∆*Mohrd3* strains respectively. The MoYpt7‐HA was detected following GFP‐MoAtg8 immunoprecipitation with or without MoHrd3‐HA. GFP: MoYpt7‐HA/P131 and GFP: MoYpt7‐HA/∆*Mohrd3* were used as negative controls. (G) The localization of GFP‐MoYpt7 and mCherry‐MoAtg8 in mycelium of P131 and ∆*Mohrd3*. Strains were cultured in liquid CM or MM‐N medium with ConA (1 µm). Mycelia were stained with CMAC to indicate the vacuoles. Scale bar, 10 µm.

To study how MoHrd3 regulates autophagy through interacting with MoAtg8, we re‐analyzed the IP‐MS data of MoHrd3‐associated proteins and found that MoYpt7, which localizes to the vacuole and is required for autophagy [23], is among the list (Table S1). We therefore assayed whether MoYpt7 interacts with MoHrd3 and MoAtg8 by yeast two‐hybrid. The result showed that MoYpt7 interacts with MoHrd3, but not with MoAtg8 (Figure 5A). The interaction between MoYpt7 and MoHrd3 was further confirmed in vivo using both co‐IP and BiFC assays (Figure 5B,C). In addition, we obtained MoHrd3‐GFP and mCherry‐MoYpt7 co‐expressed transformants to observe their co‐localization in *M. oryzae*. As shown in Figure 5D, they co‐localized on internal membranes, including the vacuolar membrane, when grown in CM medium. Upon autophagy induction in MM‐N medium, the majority of both MoHrd3‐GFP and mCherry‐MoYpt7 signals were transported into the vacuole for degradation (Figure 5D). This result demonstrates that MoHrd3 at least partially co‐localizes with MoYpt7 in *M. oryzae*.

Considering that MoHrd3 interacts with MoAtg8 and MoYpt7, respectively, we wondered whether MoHrd3 mediates the interaction between MoAtg8 and MoYpt7 to enhance the fusion of the autophagosome and the vacuole. To prove this, the interaction among them was detected using a co‐IP assay. Interestingly, unlike in yeast, MoAtg8 could interact with MoYpt7 in a co‐IP assay (Figure 5E). Notably, MoHrd3 enhances the association between MoAtg8 and MoYpt7 (Figure 5E), potentially facilitating the fusion between the autophagosome and the vacuole, and subsequently promoting autophagy. Accordingly, the interaction between MoAtg8 and MoYpt7 was significantly impaired in the ∆*Mohrd3* mutant compared to the wild‐type P131 strain (Figure 5F). This impaired interaction was effectively rescued by the addition of recombinant MoHrd3 protein in the co‐IP assay (Figure 5F). To explain this functional relationship at structure level, we further predicted the ternary complex structure of MoAtg8‐MoHrd3‐MoYpt7. AI‐based modeling suggested that MoHrd3 recruits MoAtg8 via its C‐terminal region and MoYpt7 through a large N‐terminal domain, implying that MoAtg8 and MoYpt7 bind to distinct domains of MoHrd3 to form a tripartite complex (Figure S6).

We further compared the co‐localization of MoAtg8 and MoYpt7 in P131 and ∆*Mohrd3* mutant strains. To this end, GFP‐MoYpt7 and mCherry‐MoAtg8 co‐transformants were generated in the P131 and ∆*Mohrd3* mutant strains, respectively. Under CM conditions, MoYpt7 was localized on the vacuole membrane while MoAtg8 exhibited autophagosome localization in both P131 and ∆*Mohrd3* (Figure 5G). When cultured in MM‐N medium, both mCherry‐MoAtg8 and GFP‐MoYpt7 moved into vacuoles for degradation in P131 background, while some mCherry‐MoAtg8 signal are still observed in the cytoplasm of ∆*Mohrd3* (Figure 5G), highlighting the important role of MoHrd3 in the fusion of the autophagosome and the vacuole. To determine whether MoHrd3 or MoYpt7 affects the localization of another one, the reciprocal localization assays were also detected. mCherry‐MoYpt7 was expressed in both P131 and ∆*Mohrd3* strains. Fluorescence observation revealed that the distribution of MoYpt7 was unaffected by the absence of MoHrd3 (Figure S7A). Conversely, when MoHrd3‐GFP was overexpressed in the P131 and ∆*Moypt7* backgrounds, its localization to the vacuole was abolished in the ∆*Moypt7* mutant. This result indicates that MoYpt7 is required for the vacuolar targeting of MoHrd3 (Figure S7B).

In the ∆*Mohrd3* mutant, the impaired interaction between MoAtg8 and MoYpt7 compromises autophagy, which in turn results in the reduced pathogenicity. To validate this hypothesis at the genetic level, we overexpressed MoAtg8 or MoYpt7 in the ∆*Mohrd3* mutant. As shown in Figure S7C,D, overexpression of either protein partially restored the pathogenicity of the ∆*Mohrd3* mutant. This genetic evidence further confirms that the attenuated virulence of ∆*Mohrd3* is partly due to defective autophagy.

### 2.7 MoHrd3 Mediated Autophagy is Essential for Pathogenicity of *M. oryzae*

Then we want to determine whether MoHrd3‐mediated autophagy plays a role in the pathogenicity of *M. oryzae*. A previous study showed that ER stress induced by the deletion of MoHrd1, the E3 ligase that forms a complex with MoHrd3, upregulates autophagy [18]. Given that MoHrd1 does not bind to MoAtg8 (Figure S5B), we suggest that it works in a pathway distinct from MoHrd3. Therefore, we taken MoHrd1 as a control in this infection assay. First, we obtained *MoHrd1* knockout mutants and confirmed its role in growth, conidiation, and appressoria formation (Figure S8A–E). The wound‐infection assay showed that it also participates in the regulation of pathogenicity (Figure S8F,G).

**FIGURE 6:**
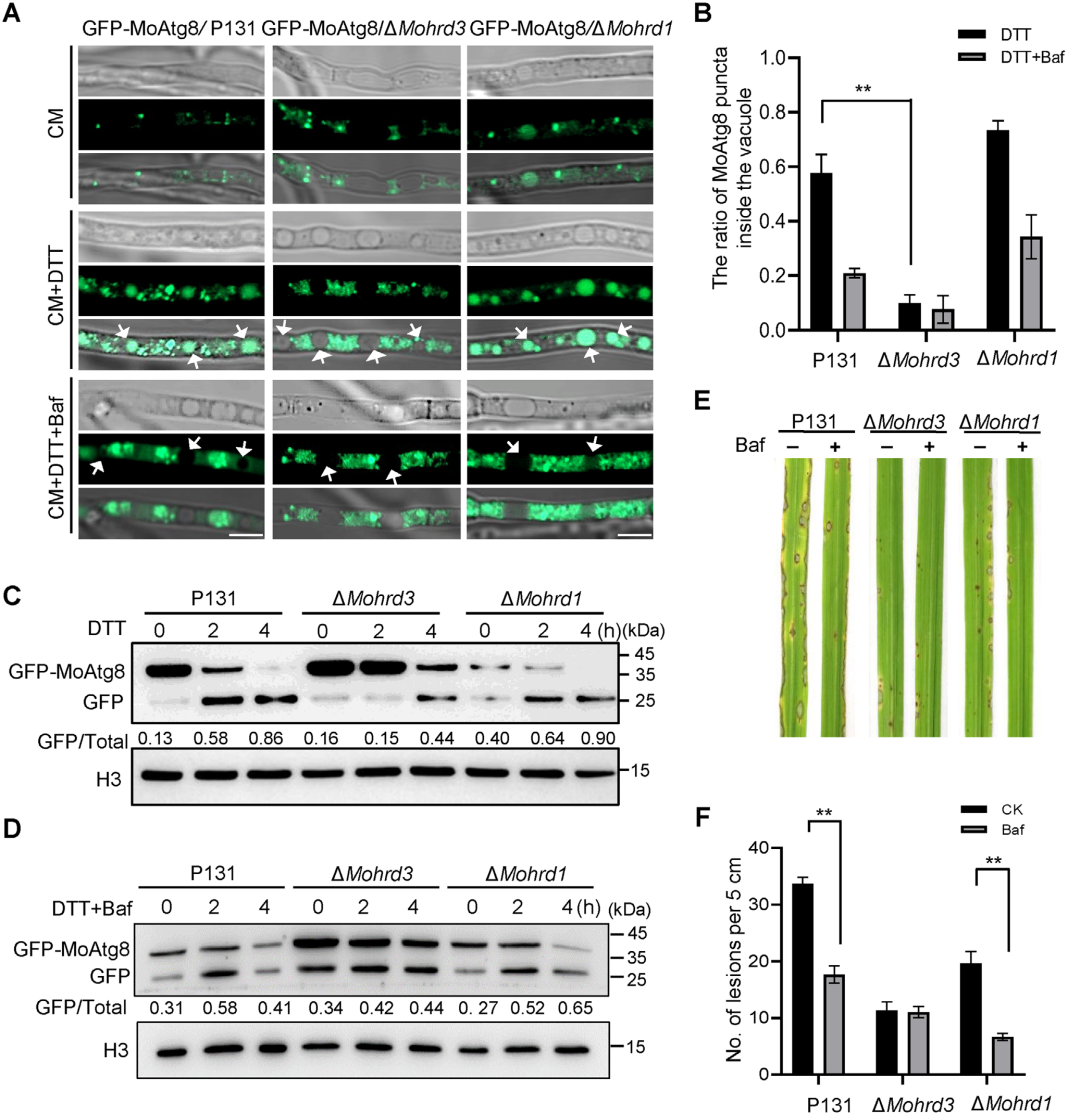
MoHrd3 participates in ER stress‐triggered autophagy. (A) The localization of GFP‐MoAtg8 in the mycelia of P131, ∆*Mohrd3*, and ∆*Mohrd1* strains. GFP‐MoAtg8/P131, GFP‐MoAtg8/∆*Mohrd3*, and GFP‐MoAtg8/∆*Mohrd1* were cultured in liquid CM for 36 h and then treated with 2 mm DTT (with or without 300 nm Baf) for 4 h before microscope observation. The white arrows indicated vacuole in hypha. Scale bar, 5 µm. (B) Quantification of GFP‐MoAtg8 puncta inside vacuoles after DTT treatment (with or without 300 nm Baf) in (A). Error bars represent SD, n = 90. The significant differences were evaluated by two‐tailed Student's *t*‐test, ^***^ *p* < 0.001. (C) The breakdown of GFP‐MoAtg8 in P131, ∆*Mohrd3*, and ∆*Mohrd1* strains after 2 mm DTT treatment. The ratio of GFP to total protein (free GFP and GFP‐MoAtg8) were calculated using Image J software. (D) The breakdown of GFP‐MoAtg8 in P131, ∆*Mohrd3*, and ∆*Mohrd1* strains after DTT (2 mm) and Baf (300 nm) treatment. The ratio of GFP to total protein (free GFP and GFP‐MoAtg8) were calculated using Image J software. (E) Rice seedlings were sprayed with conidial suspension (1.0 × 10^5^ spores/ml) of the indicated strains with or without 300 nm Baf. Photographs were taken at 5 dpi. (F) Statistical analysis of lesion numbers on rice leaves in (E). Error bars represent SD, n = 3. The significant differences were evaluated by two‐tailed Student‘s *t*‐test, **p < 0.01.

To simulate the infection‐triggered ER stress condition, we used DTT as the inducer [5]. After DTT treatment, most of GFP‐MoAtg8 in P131 and ∆*Mohrd1* were moved into the vacuole, while the GFP‐MoAtg8 signal was still localized in the cytoplasm in ∆*Mohrd3* (Figure 6A,B). Bafilomycin A1 (Baf) is a V‐ATPase inhibitor that could block the fusion between the autophagosome and the vacuole [24]. When treated with DTT and Baf together, GFP‐MoAtg8 in P131 and ∆*Mohrd1* are also detained in cytoplasm, similar to what was observed in ∆*Mohrd3* (Figure 6A,B). We also assessed the autophagic flux by analyzing the breakdown of GFP‐MoAtg8 under DTT treatment. DTT normally triggered the autophagy process in P131 and ∆*Mohrd1*, while the DTT‐triggered autophagy level is largely reduced in ∆*Mohrd3* (Figure 6C). Following treatment with DTT and Baf together, autophagy was blocked in P131 and both mutants (Figure 6D). Importantly, the interaction between MoAtg8 and MoHrd3 is enhanced by DTT treatment (Figure S9A). Above results demonstrate that *M. oryzae* infection could induce autophagy, and MoHrd3 plays an important role in infection‐triggered autophagy.

We further examined the effect of Baf in pathogenicity using rice spray‐and wound‐inoculation methods, respectively. In the spray assay, Baf treatment could inhibit the pathogenicity of both P131 and ∆*Mohrd1*. However, in ∆*Mohrd3* mutant, its pathogenicity did not further reduce by Baf treatment (Figure 6E,F). The wound‐infection assay also obtained a similar result (Figure S9B,C), supporting that MoHrd3‐mediated fusion between the autophagosome and the vacuole is important for pathogenicity in *M. oryzae*.

### 2.8 MoHrd3 Acts as an Adaptor Protein to Promote the Autophagic Degradation of the GPCR Protein MoPth11

Previous results showed that MoHrd3 binds to MoAtg8 directly and itself could be degraded through the autophagy pathway. These clues were in line with the selective autophagy receptor, and thus we performed a yeast two‐hybrid assay to confirm whether MoHrd3 interacts with MoAtg8 through an AIM motif. Through online prediction tools (https://ilir.warwick.ac.uk/search.php), 10 AIM motifs in MoHrd3 were identified (Figure S10A). Among them, three high scored sites, including amino acid sequence 266‐271, 302‐307, and 686‐691, were submitted to further validation (Figure S10B). Unfortunately, when all these three high scored AIM motifs were mutated through point mutation, the interaction between MoHrd3 and MoAtg8 was still observed, indicating that their interaction may be AIM‐independent (Figure S10C). Considering that only the C‐terminal of MoHrd3 is localized in the cytoplasm, we examined the role of MoHrd3's C‐terminal in its association with MoAtg8. The results of yeast two‐hybrid and co‐IP assays showed that MoAtg8 could not bind to the C‐terminal deleted MoHrd3 (Figure S10D,E), suggesting that MoHrd3 associates with MoAtg8 through its C‐terminal. Therefore, MoHrd3 is not a typical selective autophagy receptor in *M. oryzae*.

**FIGURE 7:**
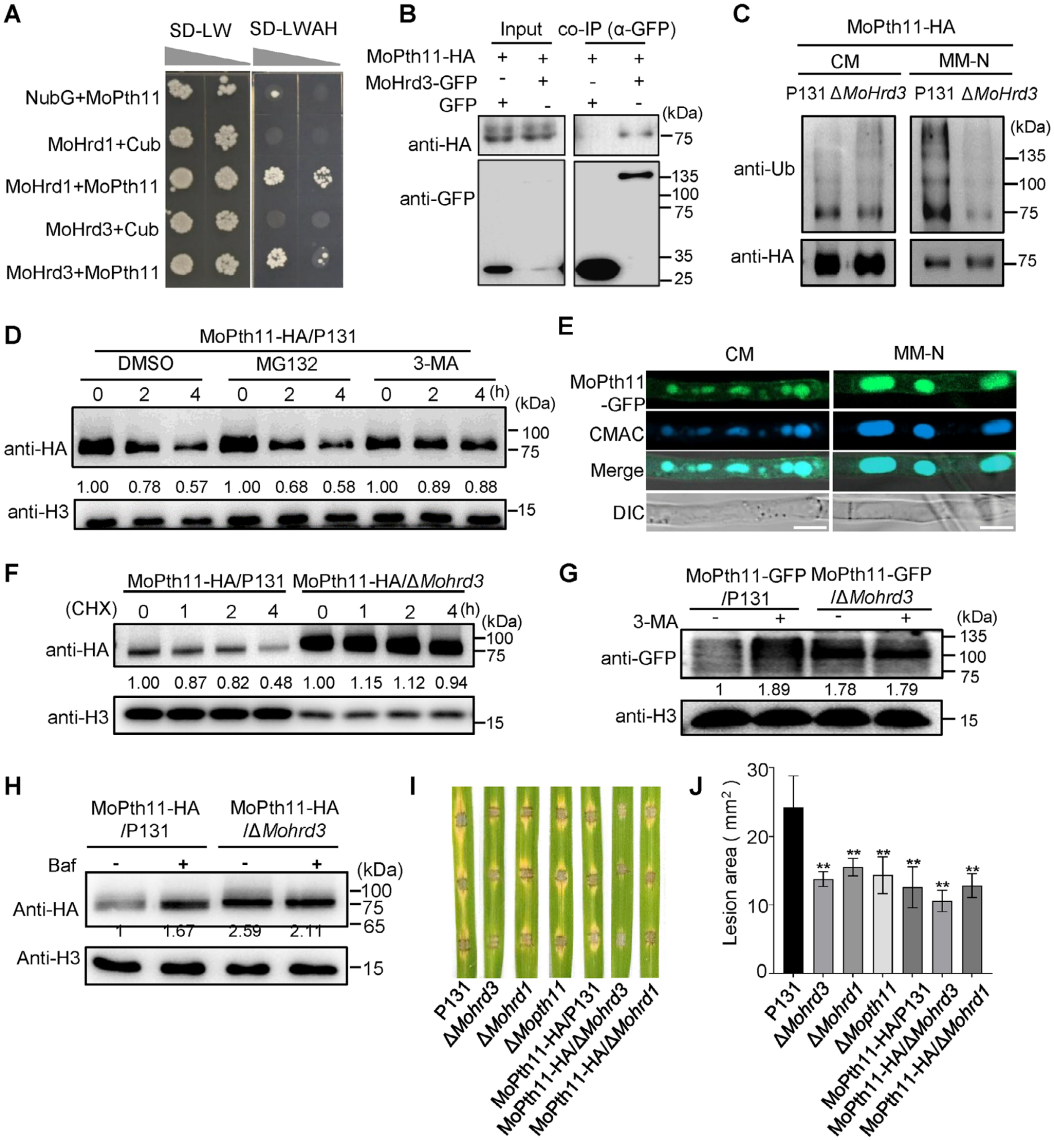
MoHrd3 facilitates the autophagic degradation of MoPth11. (A) MoHrd1 and MoHrd3 interacted with MoPth11 in yeast. (B) MoHrd3 interacted with MoPth11 in a co‐IP assay. MoPth11‐HA/P131 and MoHrd3‐GFP/P131 strains were used in this assay. MoPth11‐HA was detected following MoHrd3‐GFP immunoprecipitation. (C) The ubiquitination level of MoPth11 in the MoPth11‐HA/P131 and MoPth11‐HA/Δ*Mohrd3* was assayed using ubiquitin antibody. Total protein was subjected to HA‐immunoprecipitation and then assessed using the indicated antibodies. All strains were cultured in liquid MM‐N medium for 5 h. (D) Protein stability of MoPth11 upon treatment with different inhibitors. Strains were cultured in liquid CM for 36 h and then treated with DMSO, MG132 (100 µm) or 3‐MA (500 µm) for 0, 2, and 4 h, respectively. (E) MoPth11‐GFP was localized to the vacuole in response to MM‐N treatment. MoPth11‐GFP/P131 stain was cultured in liquid CM for 36 h and then treated with MM‐N for 5 h. All strains were treated with 0.1 g/ml LatB for 1 h before microscope observation. Mycelia were stained with CMAC to indicate the vacuoles. Scale bar, 5 µm. (F) MoPth11 is accumulated in ∆*Mohrd3* mutant. Strains were treated with 30 µm CHX for 0, 1, 2, 4 h. (G,H) MoHrd3 is required for the autophogic degradation of MoPth11. All strains were cultured in liquid CM medium with or without (G) 500 µm 3‐MA or (H) 300 nm Baf for 4 h. (I) Rice leaves were drop‐inoculated with conidial suspensions (1 × 10^5^ spores/ml) of the indicated strains, and photographs were taken at 5 dpi. (J) Statistical analysis of lesion area on rice leaves in (I). Error bars represent SD, n = 6. The significant differences were evaluated by two‐tailed Student's *t*‐test, ^**^ *p* < 0.01.

In ERAD pathway, Hrd3 serves as an adaptor protein that recognizes target proteins for proteasome‐mediated degradation. This prompted us to investigate whether MoHrd3 also acts as an adaptor protein to mediate the autophagic degradation of its targets. MoPth11, an atypical G protein‐coupled receptor (GPCR), is essential for appressoria formation and pathogenicity in *M. oryzae* and is transported from plasma membrane (PM) to ER through the endocytosis pathway [25, 26]. Considering that MoHrd3 also regulates appressoria formation and pathogenicity, we tested whether MoPth11 works as a target protein of MoHrd3 and participates in MoHrd3‐mediated pathogenicity. First, we confirmed that MoPth11 interacts with MoHrd3, as well as MoHrd1, the E3 ligase that forms a complex with MoHrd3 (Figure 7A). Their interaction was further confirmed using co‐IP assays (Figure 7B; Figure S11A).

To determine whether MoPth11 acts as a target of MoHrd3, we detected the ubiquitination and protein accumulation levels of MoPth11 in P131 and ∆*Mohrd3*. After purification using anti‐HA agarose, the products were detected using anti‐ub and anti‐HA antibodies. The results showed that a much reduced ubiquitination level of MoPth11 was shown in ∆*Mohrd3* under starvation conditions (MM‐N treatment), while similar ubiquitination levels were detected under normal conditions (CM media), These observations indicated that MoHrd3 is required for starvation induced MoPth11 ubiquitination (Figure 7C). To determine the degradation pathway of MoPth11, P131 was cultured in CM medium for 36 h and then treated with DMSO (control), MG132 (a 26S proteasome inhibitor), or 3‐MA (an autophagy inhibitor). As shown in Figure 7D, the degradation of MoPth11 was suppressed by 3‐MA but not by MG132. Furthermore, MoPth11‐GFP localized to the vacuole in mycelium cultured in MM‐N medium (Figure 7E), collectively indicating that MoPth11 could be degraded via the autophagy pathway. Notably, the autophagic degradation of MoPth11 was abolished in the ∆*Mohrd3* mutant (Figure 7F). When block the endocytosis pathway using Latrunculin B (LatB) treatment [25], a small fraction of MoPth11 localized to PM, while its PM localization in ∆*Mohrd3* mutant is clearly enhanced, suggesting that MoHrd3 is required for the autophagic degradation of MoPth11 (Figure S11B). Consistent with this finding, treatment with the autophagy inhibitors 3‐MA and Baf increased MoPth11 accumulation in P131, whereas MoPth11 levels remained constitutively high in the ∆*Mohrd3* mutant regardless of inhibitor treatment (Figure 7G,H). qPCR analysis confirmed that *MoPth11* mRNA levels were similar among all strains (Figure S11C,D), ruling out the regulation at transcriptional level. Similar to MoHrd3, MoHrd1 was also required for starvation‐induced ubiquitination (Figure S11E) and accumulation of MoPth11 (Figure S11F–I). Collectively, these results indicate that MoPth11 is degraded through autophagy following ubiquitination mediated by the MoHrd1‐MoHrd3 complex.

Previous studies on MoPth11 reported that loss function of *MoPth11* results in reduced pathogenicity of *M. oryzae* [27]. To determine whether MoPth11 involves in MoHrd3 mediated pathogenicity, we compared the pathogenicity of P131, ∆*Mohrd3*, ∆*Mohrd1*, ∆*Mopth11*, and *MoPth11* overexpression transformants in the ∆*Mohrd3* and ∆*Mohrd1* mutant strains (Figure S11J). As shown in Figure 7I,J, MoPth11 overexpression transformants in P131 exhibited reduced pathogenicity, suggesting the reduced pathogenicity of ∆*Mohrd3* may be partially caused by the accumulated MoPth11 protein. The observation that the MoPth11 overexpression transformant exhibited a phenotype similar to the ∆*Mopth11* mutant is consistent with previous findings, which demonstrated that constitutive expression of the CFEM domain (crucial for MoPth11 function) results in reduced pathogenicity [28]. It demonstrates that maintaining an appropriate level of MoPth11 may be essential for the pathogenicity of *M. oryzae*. However, the underlying mechanism should be explored further in future research.

## 3 Discussion

**FIGURE 8:**
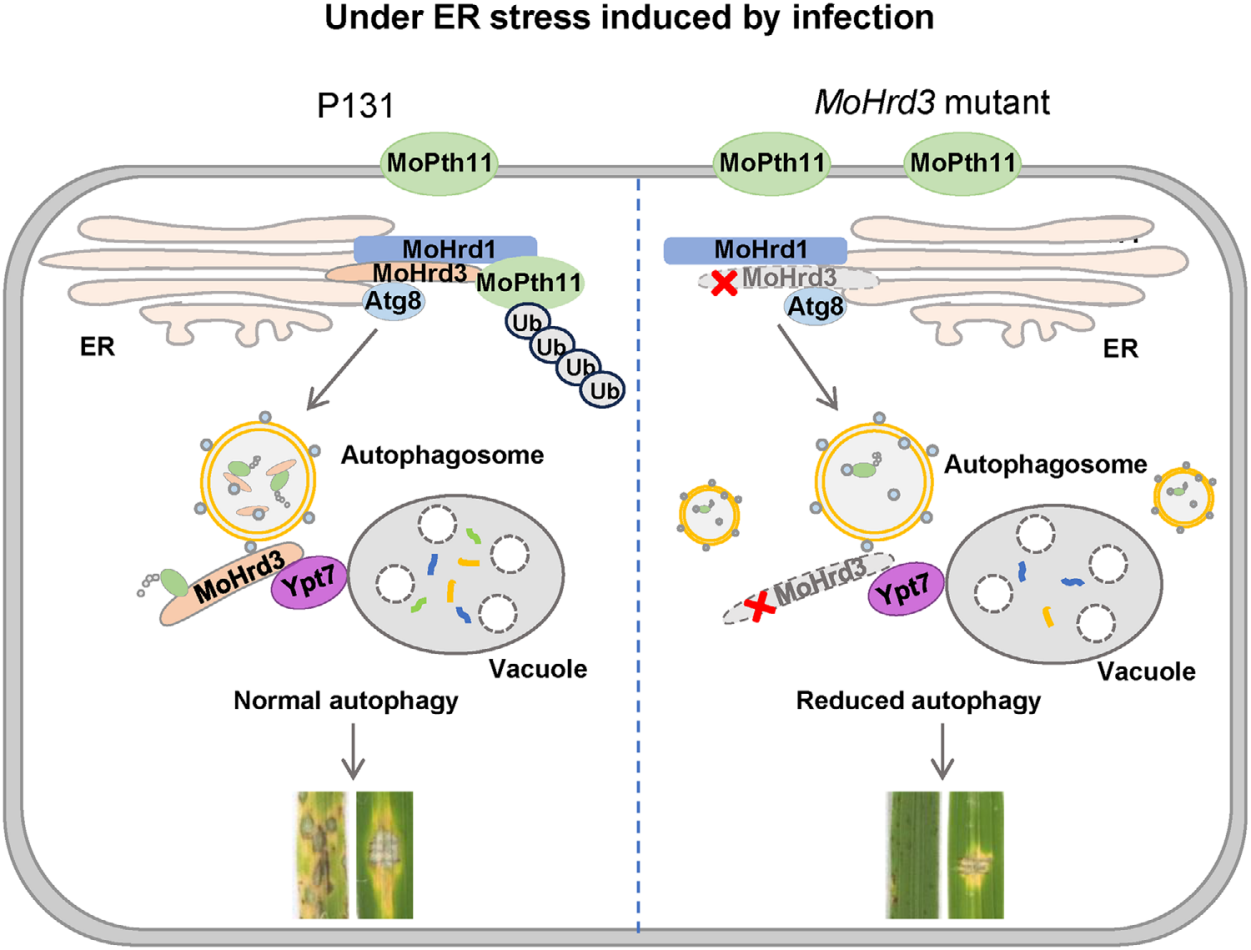
The working model of MoHrd3 in the pathogenicity of *M. oryzae*. Infection induces ER stress, which subsequently trigger autophagy. MoHrd3, an ERAD component, involves in ER stress‐triggered autophagy through enhancing the fusion between autophagosome and vacuole. Additionally, the MoHrd1‐MoHrd3 complex mediates the ubiquitination of MoPth11. MoHrd3 then serves as an adaptor protein to facilitate the autophagic degradation of MoPth11. In the absence of *MoHrd3* (∆*Mohrd3* mutant), the fusion between autophagosome and vacuole is severely compromised, resulting in reduced autophagy and the accumulation of MoPth11 (as well as other unknown targets). This leads to a decrease in the pathogenicity of *M. oryzae*.

ERAD and autophagy are two main strategies to maintain ER homeostasis. Although they have been reported to be closely related, the direct regulation mechanism behind them is largely unknown. In this study, we found that MoHrd3, an ERAD component that is essential to the pathogenicity and appressorium formation of *M. oryzae*, regulates autophagy levels by promoting the fusion of the autophagosome and the vacuole. Further study showed that MoHrd3 interacts with both MoAtg8 and MoYpt7, and works as an adaptor protein to promote the association between MoAtg8 and MoYpt7, finally promotes the progress of infection‐triggered autophagy. In addition, MoHrd3 acts as an adaptor protein that binds to MoAtg8 through its C‐terminal domain. It mediates the autophagic degradation of MoPth11, following the ubiquitination modification mediated by the MoHrd1‐MoHrd3 complex, to regulate the pathogenicity of *M. oryzae*. MoHrd3 itself could also be degraded through the autophagy pathway. Moreover, the increased MoPth11 protein level in ∆*Mohrd3* mutant also results in reduced pathogenicity of *M. oryzae* (Figure 8).

According to the study in yeast, mammals, and plants, ERAD components regulate development, stress response, and metabolic process mainly by targeting key factors in these signaling pathways [5, 29]. However, the research about ERAD in plant pathogenic fungi is largely limited. Although several ERAD components, including MoHrd1, MoDer1, and MoCue1, have been proven to regulate the pathogenicity of *M. oryzae* by analyzing the virulence phenotype of gene knockout mutants, most of the detailed mechanism is absent [18, 30]. In this study, we identified a new ERAD component, MoHrd3 that affects the pathogenicity and appressorium formation of *M. oryzae*, as well as the autophagy level. Further study showed that MoHrd3 regulates autophagy by facilitating the fusion of the autophagosome and the vacuole, a process that is essential for pathogenicity. Therefore, our study firstly demonstrated the important role of ERAD component in the autophagy process in *M. oryzae*. It is noteworthy that, although MoAtg8 and MoYpt7 did not interact directly in yeast, their interaction was still detectable via co‐IP in the ∆*Mohrd3* mutant. This suggests that other adaptor proteins, such as SNAREs, may function redundantly or alternatively to mediate the association between MoAtg8 and MoYpt7 in the absence of MoHrd3.

Several examples about the regulation between ERAD and autophagy have been demonstrated in mammals. ERAD‐related E3 ligase Gp78/Autocrine Motility Factor Receptor (AMFR) is degraded through the autophagy pathway, which is mediated by the reticulophagy receptor and regulator (RETREG1) [24]. A recent study showed that AMRF catalyses the ubiquitination of ER‐phagy receptor FAM134B, a mammalian reticulon‐like protein that shapes the ER membrane, thereby positively regulating ER‐phagy [31]. Also in mammal, another E3 ligase HRD1 promotes the degradation of the SERPINA1 Z variant/α‐1‐antitrypsin Z variant through SQSTM1/p62 mediated selective autophagy [17]. Taken together, ERAD components regulate selective autophagy mainly by modifying the receptor or cargo. In this study, we proved that MoHrd3acts as an adaptor protein, although not a typical selective autophagy receptor, in autophagy. It binds to MoAtg8 directly and recruits MoPth11 as a target protein to regulate the pathogenicity of *M. oryzae*. Therefore, our data support that ERAD components directly participate in the autophagy process.

The G‐protein‐coupled receptors (GPCRs) are cell‐surface proteins with seven transmembrane α‐helices that recognize ligands and then transfer the signal cues through G proteins conformational change [32]. There are 76 GPCR‐like proteins in *M. oryzae*, and MoPth11 is the first characterized GPCR in *M. oryzae* [28, 33]. MoPth11 has been shown to be required for appressorium formation and virulence [33]. Here we found that the high MoPth11 level also reduces pathogenicity, like what has been observed in *MoPth11* mutant. This observation is consistent with the previous result, in which constitutive expression of the CFEM domain that is important for MoPth11 function, results in reduced pathogenicity [27]. Above results demonstrate that an appropriate MoPth11 protein level is important for pathogenicity of *M. oryzae*. Coincidentally, we found the stability of MoPth11 is regulated by autophagy in MoHrd3‐MoHrd1 complex‐dependent manner. Considering that MoHrd3 and MoHrd1 also promote proteasome‐mediated degradation, more target proteins that recruits by MoHrd3 should be characterized in the future to study what determines a target protein degraded through the proteasome pathway or the autophagy pathway.

## 4 Experimental Methods

### 4.1 Strains and Culture Conditions

The *M. oryzae* P131 (field isolate) was used as the wild‐type strain in this study. For vegetative growth and conidia quantity assays, different strains were grown on oatmeal tomato agar (OTA) medium for 5 days at 28°C. For experiments on TM treatment, 5 mm mycelial blocks of P131, ∆*Mohrd3* and *cMoHrd3* strains were placed on complete medium (CM) plates (6 g yeast extract, 3 g casein acid hydrolysate, 3 g casein enzymatic hydrolysate, and 10 g sucrose in 1 L of distilled water) with or without 0.2 µg/mL TM (MedChemExpress) and grown at 28°C for 5 days. For DNA, RNA, protein extractions and isolation of protoplasts, strains were initially grown on OTA medium at 28°C for 7 days and then transferred to liquid CM medium for another 36 h incubation before harvest.

### 4.2 Gene Knockout and Complementation

To generate *MoHrd3* deletion mutants, the upstream and downstream 1.5 kb sequence fragments of *MoHrd3* were amplified from P131 genomic DNA and cloned into pKOV21 vector. The obtained plasmid was delivered into the protoplasts of P131 to obtain *MoHrd3* targeted knockout mutants. ∆*Mohrd3* mutants were confirmed by PCR and Southern blot assays. For gene complementation of the ∆*Mohrd3* mutant, the full‐length genomic sequence of *MoHrd3* was amplified and cloned into the pGTN vector (contain a GFP tag at the C‐terminal) with 2 kb native promoter to generate MoHrd3‐GFP construct. The MoHrd3‐GFP plasmid was transformed into ∆*Mohrd3* mutant. All neomycin‐resistant transformants were further confirmed by western blot with anti‐GFP antibody and phenotypic analysis. The primers used to in this study are listed in Table S2. The *MoHrd1* gene deletion mutants (∆*Mohrd1*) and the *MoHrd1* complementation strains (*cMoHrd1*) were obtained using a similar strategy.

### 4.3 Southern Blot Analysis

EcoR I was used to digest the genomic DNA of P131 and the Δ*Mohrd3* mutants. The digest products were separated in 1.0% agar gel and were hybridized with the indicated probe. In P131, a 5894 bp fragment was detected by the probe, while the digestion production is 4599 bp in Δ*Mohrd3*. The whole hybridization was carried out according to the manufacturer's instruction for DIG‐High Prime (Roche, 11745832910). The same method was used to confirm the knockout mutants of *MoHrd1*.

### 4.4 Pathogenicity, the Growth of Infection Hyphal and Appressorium Formation Assays

The pathogenicity assay was performed as described previously [34]. In brief, rice seedlings at the fourth leaf stage were inoculated with different *M. oryzae* strains using the drop or spray inoculation methods. Rice seedlings were sprayed or dropped with conidia suspension of 1.5 × 10^5^ spores/mL in 0.025% Tween 20 solution with or without 300 nm Baf (Bafilomycin A1, MedChemExpress). Number of lesions or lesion area on leaves were calculated after incubated for 5 days at 28°C.

To investigate the infection process in plant cell, a conidial suspension with a concentration of 1.5 × 10^5^ spores/mL in 0.025% Tween 20 solution was injected into the detached rice sheaths, followed by incubation at 28°C, and then incubated in a moist. The infected epidermis was observed using a Nikon Ni 90 microscope at 36 hpi and 48 hpi.

In appressorium formation assay, conidial suspension (1 × 10^5^ spores/mL) was inoculated on hydrophobic coverslips (VWR, 48366‐067) in the dark for 6 h at 28°C and the appressorium formation was then observed under a Nikon Ni 90 microscope.

### 4.5 RT‐qPCR

Total RNA was isolated by KK Fast Plant Total RNA Kit (Beijing Zoman Biotechnology, ZP401) according to the manufacturer's protocol and was reverse‐transcribed by HiScript III first Strand cDNA Synthesis Kit (Vazyme Biotech Company) with gDNA. *Actin* gene was used as a control. Gene‐specific primers are presented in Table S2.

### 4.6 Subcellular Localization Analysis

In order to observe the subcellular localization of MoHrd3, pEF1α‐MoHrd3‐GFP (driven by the promoter of EF1a [MGG_03641]) and RP27‐mCherry‐HDEL (the ER marker) were co‐transformed into the protoplasts of P131. For BiFC assay, pEF1α‐MoAtg8‐nYFP or pEF1α‐MoYpt7‐nYFP plasmid was co‐transformed with pEF1α‐MoHrd3‐GFP into the protoplasts of the Δ*Mohrd3* mutants. Hyphae were treated with or without 1 µm ConA. For co‐localization analysis of MoHrd3 and MoAtg8, pEF1α‐MoHrd3‐GFP and RP27‐mCherry‐MoAtg8 plasmids were co‐transformed into the protoplasts of P131. Hyphae were stained with 20 µm CMAC (7‐amino‐4‐chloromethylcoumarin, Invitrogen), a dye that labels the lumen of fungal vacuoles (InvivoChem, V49311). For co‐localization analysis of MoHrd3 and MoYpt7, pEF1α‐MoHrd3‐GFP and native promoter‐driven GFP‐MoYpt7 were co‐transformed into P131 strain. Stain was cultured in liquid CM medium with or without further treatment with MM‐N medium (with 1 µm ConA) for 5 h before observation. CMAC was used to mark vacuoles. For co‐localization analysis of MoYpt7 and MoAtg8, native promoter‐driven GFP‐MoYpt7 and RP27‐mCherry‐MoAtg8 plasmids were co‐transformed into the protoplasts of the P131 and Δ*Mohrd3* mutant. The transformed strains were cultured in liquid CM medium for 36 h at 28°C, and then shifted to fresh liquid MM‐N medium with or without 1 µm ConA (Concanamycin A, MedChemExpress) for 5 h before the observation. Sep3‐GFP and Sep5‐GFP plasmids were transformed into the P131 strain and the Δ*Mohrd3* mutant, respectively, for the septin ring formation assay. The samples were observed under fluorescence microscopy (Zeiss, LSM 800). For the subcellular localization of MoPth11, pEF1α‐MoPth11‐GFP was transformed into the protoplasts of P131 and Δ*Mohrd3*. Strains were cultured in liquid CM for 36 h and then treated with or without MM‐N for 5 h. All strains were treated with 0.1 g/mL Latrunculin B for 1 h (MCE, HY‐101848) before microscope observation.

### 4.7 Appressorium Turgor Determination and Glycogen/Lipid Staining

Appressorium turgor was determined by cell collapse assay using a 0‐3 M concentration of glycerol solution. The samples were observed under a Nikon Ni 90 microscopy. For glycogen and lipid droplet staining assay, the conidial suspension (1×10^5^ spores/mL) was inoculated on hydrophobic coverslips in the dark for 0, 6, 8, 12, and 24 h, respectively. The conidia were stained with KI/I_2_ solution (60 mg/mL KI, 10 mg/mL I_2_) for 1 min or Nile red solution (50 mM Tris/maleate buffer, 20 mg/mL polyvinylpyrrolidone, 2.5 µg/mL Nile red, pH 7.5) for 3 min.

### 4.8 Yeast Two‐Hybrid Assays

The split‐ubiquitin membrane yeast two‐hybrid (Y2H) system was used in this study. The Y2H assays were performed according to the manufacturer's protocol of the DUAL membrane kit 3 (DUAL systems, BioTech). The full‐length *MoHrd3* coding sequence was cloned into pBT3‐STE to generate MoHrd3‐Cub, while the full‐length *MoHrd1*, *MoAtg8*, *MoYpt7*, or *MoPth11* coding sequence were cloned into pPR3N. The constructs and their control vectors were co‐transformed into the NMY51 strain using the PEG/LiAc method. The colonies grown on synthetic defined (SD) medium without Leu and Trp were transferred onto SD/‐Leu‐Trp‐His‐Ade selection medium with 3‐amino‐1, 2,4‐triazole (3‐AT, Coolaber) to confirm the protein‐protein interaction.

### 4.9 Co‐Immunoprecipitation (co‐IP) Assays

pEF1α‐MoHrd3‐GFP/P131, pEF1α‐MoHrd3‐HA/P131, pEF1α‐MoHrd3(∆C)‐HA/P131, RP27‐GFP‐MoAtg8/P131, pEF1α‐ GFP‐MoYpt7/P131, pEF1α‐MoYpt7‐HA/P131, pEF1α‐MoPth11‐GFP/P131, and pEF1α‐MoPth11‐HA/P131 transformations were generated for co‐IP assays. To detect the influence of MoHrd3 on the interaction between MoYpt7 and MoAtg8, GFP or GFP‐MoAtg8 plasmids were co‐transformed with MoYpt7‐HA plasmid in P131 or ∆*Mohrd3* strains, respectively. GFP: MoHrd3‐HA/∆*Mohrd3* and GFP‐MoAtg8: MoHrd3‐HA/∆*Mohrd3* strains were generated to determine the interaction between MoAtg8 and MoHrd3 under DTT treatment. Total proteins were extracted from mycelia using the protein lysis buffer (20 mM Tris‐HCl, pH 7.5, 150 mM NaCl, 0.5 mM EDTA, 0.5% NP‐40) with 4 mm PMSF and 1 mm DTT. Proteins were extracted and incubated with 15 µL of prewashed anti‐GFP Nanobody Magarose Beads (AlpaLifeBio) or anti‐HA magnetic beads (MedChemExpress) at 4°C for 3 h. The beads were collected by centrifugation and then washed three times with cold PBS buffer (containing 1% PMSF). The samples were separated by SDS‐PAGE and detected by immunoblotting with anti‐GFP (ABclonal, 1: 5,000) and anti‐HA (ABclonal, 1: 10,000) antibodies.

### 4.10 Protein Degradation Assays

The MoHrd3‐HA/P131 transformed strain was cultured in liquid CM medium for 36 h at 28°C, and then treated with 10 mm DTT (Coolaber), 100 µM MG132 (MedChemExpress), or 500 µm 3‐MA (MedChemExpress) for 10 h. The MoPth11‐GFP/P131, MoPth11‐GFP/Δ*Mohrd3*, and MoPth11‐GFP/Δ*Mohrd1* transformed strains were cultured in liquid CM medium for 36 h at 28°C, and then treated with MG132 (100 µm), 3‐MA (500 µm), or Baf (300 nm). Proteins were detected by immunoblotting with anti‐H3 (EASYBIO, 1: 5,000), anti‐HA, and anti‐GFP antibodies.

### 4.11 Autophagy Assays

In order to detect the autophagy process, the RP27‐GFP‐MoAtg8/P131 and RP27‐GFP‐MoAtg8/Δ*Mohrd3* transformed strains were cultured in liquid CM medium for 30 h at 28°C, and then shifted to fresh liquid MM‐N medium (10 g glucose, 0.52 g KCl, 0.52 g MgSO_4_, 1.52 g KH_2_PO_4_, 0.5% biotin dissolved in 1 L of ddH_2_O, pH 6.5) with 2 mM PMSF for 2 h and 4 h to induce autophagy. GFP‐Atg8 degradation was probed with anti‐GFP. The ratio of free GFP to total protein (free GFP and GFP‐MoAtg8) was calculated using Image J software. The localization of GFP‐MoAtg8 in P131 and Δ*Mohrd3* was observed after being cultured in MM‐N medium for 5 h. The localization of GFP‐MoAtg8 in P131, Δ*Mohrd3*, and Δ*Mohrd1* were observed after being cultured in CM medium with 2 mm DTT (with or without 300 nmBaf) for 4 h. Mycelia were stained with CMAC to indicate the vacuoles. The autophagosomes in the P131 and Δ*Mohrd3* strains were observed using scanning electron microscope (HITACHI, Ruli TEM, HT7800). The detection of endogenous MoAtg8/MoAtg8‐PE was performed according to the previous study [35]. The P131 and Δ*Mohrd3* strains were cultivated in CM for 36 h and then transferred to MM‐N medium for 4 h. Proteins were isolated using the TCA method and analyzed by western blotting (13.5% SDS‐PAGE in the presence of 6 M urea [Sigma–Aldrich, U5128]). MoAtg8 proteins were detected by immunoblotting with antiAtg8 [1:2000, MBL Beijing Biotech; PM090].

### 4.12 In Vivo Ubiquitination Assays

For total protein ubiquitination level detection, P131 and *∆Mohrd3* strains were cultured in liquid CM medium for 36 h at 28°C. In order to detect the MoPth11 proteins ubiquitination level, the MoPth11‐GFP/P131, MoPth11‐GFP/Δ*Mohrd3*, and MoPth11‐GFP/Δ*Mohrd1* transformed strains were cultured in liquid CM medium for 36 h at 28°C, and then shifted to MM‐N medium or treated with 10 mm DTT for 4 h. 1 µg ubiquitin was added to the protein extract before being incubated with 20 µL of prewashed anti‐HA magnetic beads at 4°C for 3 h. Proteins were detected by immunoblotting with anti‐ubiquitin antibody (EASYBIO, 1: 1000).

### 4.13 Statistics Analysis

Quantitative data are presented as the mean ± standard deviation (SD) and were analyzed using GraphPad 8. Significant differences between treatments were statistically determined by Student's *t*‐test (^*^ *p* < 0.05, ^**^ *p* < 0.01, and ^***^ *p* < 0.001).

### 4.14 Accession Numbers

Genes involved in this article can be found in the GenBank database under the following accession numbers: MoHrd3 (MGG_13508), MoAtg8 (MGG_01062), MoYpt7 (MGG_08144), MoPth11 (MGG_05871), MoHrd1 (MGG_09205).

## Author Contributions

Qian Chen, Huiqing Xia, and Yunna Zheng conceived the study, and designed all experiments and analyzed the data. Huiqing Xia performed most subcellular localization analysis, co‐IP assays and in vivo ubiquitination assays. Gene knockout and some phenotypes were performed by Yunna Zheng. Dongli Wang helps in AI based complex model. Nan Yang, Jing Chen, Xi Wu, Danrui Cui, and Ximing Zheng helps in some western blots and phenotypes. Ruijin Wang and Xunli Lu assisted in the design, analysis, and interpretation of experiments. All authors discussed the results and Qian Chen, You‐liang Peng, and Huiqing Xia wrote the manuscript with comments from other authors.

## Conflicts of Interest

The authors declare no conflicts of interest.

## Supporting information




**Supporting File 1**: advs74457‐sup‐0001‐SuppMat.pdf.

**Supporting File 2**: advs74457‐sup‐0002‐Table S1.docx.

**Supporting File 3**: advs74457‐sup‐0003‐Table S2.docx.

## Data Availability

The data that support the findings of this study are available from the corresponding author upon reasonable request.
