# Supplementary material for: ERAD Component MoHrd3 Facilitates Pathogenicity and Establishes a Direct Regulation on Autophagy in Magnaporthe Oryzae
Source: Adv Sci (Weinh). 2026 Feb 23;13(24):e20627. doi: 10.1002/advs.202520627 (PMC13116320; doi:10.1002/advs.202520627)
Supplement: Supplementary file 1 — Supporting File 1: advs74457‐sup‐0001‐SuppMat.pdf. [file ADVS-13-e20627-s003.pdf]

Figure S1

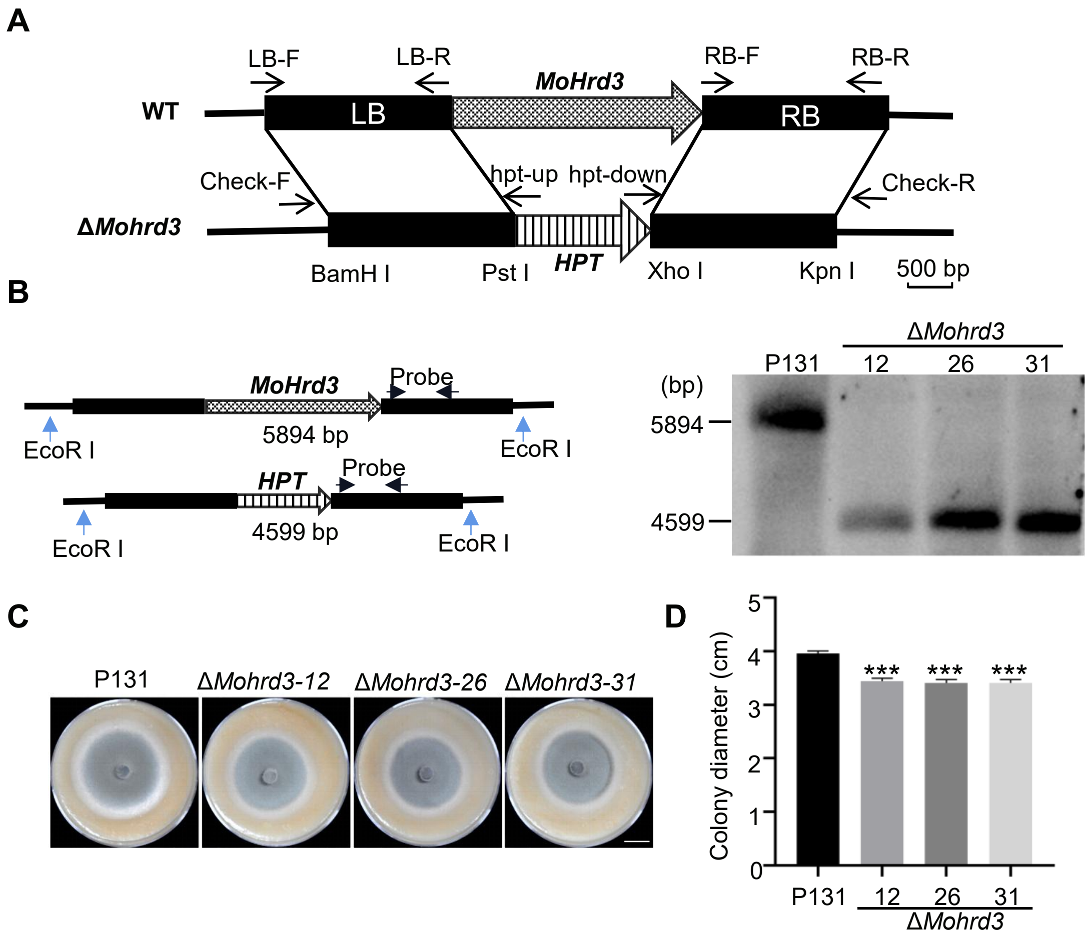

Figure S1. Gene knockout of *MoHrd3* and phenotypic analysis of the *MoHrd3* mutant. A) Schematic diagram of the *MoHrd3* deletion strategy. *HPT*, hygromycin phosphotransferase gene. B) Southern blot analysis of the *MoHrd3* deletion mutants. EcoR I was used to digest the genomic DNA of P131 and three *MoHrd3* candidate mutants. The probe was amplified from DNA of P131. C) and D) Colony diameter was measured at 5 days on OTA plates. Scale bP131 and three *MoHrd3* candidate mutants (number in 12, 26 and 31) were used in this assayar: 1 cm. Error bars represent SD, n = 3. The significant differences were evaluated by two-tailed Student's *t*-test, ***$p < 0.001$.

Figure S2

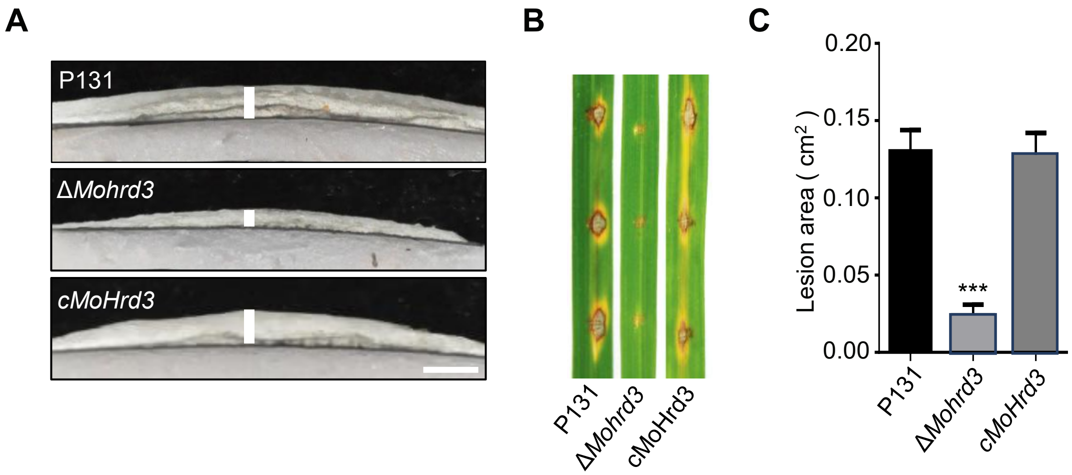

Figure S2. MoHrd3 is important for hyphal growth and pathogenicity in *M.oryzae*. A) Hyphal growth of P131, Δ*Mohrd3* and *cMoHrd3* cultured at 28ºC for 5 days. Scale bar: 5 mm. B) Wounded rice leaves were drop-inoculated with conidial suspensions (1.5×10^5 spores/ml) of the indicated strains and photographs were taken at 5 dpi. C) Statistical analysis of lesion area on rice leaves in (B). Error bars represent SD, n = 9. The significant differences were evaluated by two-tailed Student's *t*-test, ***$p < 0.001$.

Figure S3

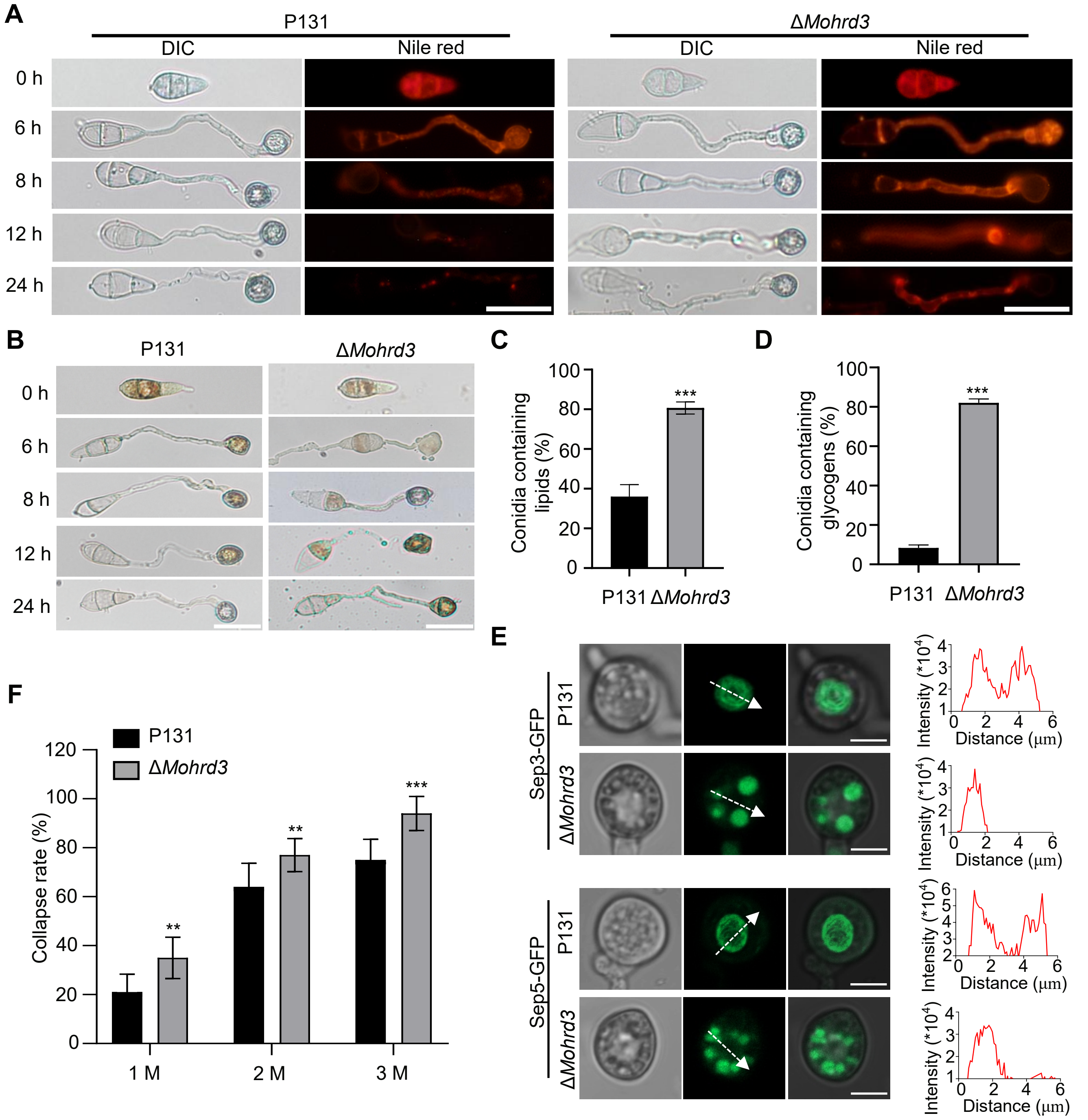
A
P131
ΔMohrd3
DIC
Nile red
DIC
Nile red
0 h
6 h
8 h
12 h
24 h
B
P131
ΔMohrd3
0 h
6 h
8 h
12 h
24 h
C
Conidia containing lipids (%)
100
80
60
40
20
0
***
P131
ΔMohrd3
D
Conidia containing glycogens (%)
100
80
60
40
20
0
***
P131
ΔMohrd3
E
Sep3-GFP
P131
ΔMohrd3
Sep5-GFP
P131
ΔMohrd3
Intensity (*10⁴)
Distance (μm)
F
P131
ΔMohrd3
Collapse rate (%)
120
100
80
60
40
20
0
**
**
***
1 M
2 M
3 M

Figure S3. *MoHrd3* is required for the maturation of appressoria. A) Glycogen distribution during the development of appressoria in P131 and Δ*Mohrd3*. Scale bar, 20 μm. B) Cellular distribution of lipid droplets during the development of appressoria in P131 and Δ*Mohrd3*. Scale bar: 20 μm. C, D) Proportion of the conidial cells containing glycogen stained by KI/I_2 solution and lipids stained by Nile red during appressorium development in the P131 and Δ*Mohrd3* mutant. Error bars represent SD, n = 300. The significant differences were evaluated by two-tailed Student's *t*-test, *** $p < 0.001$. E) The localization of Sep3-GFP and Sep5-GFP in P131 and Δ*Mohrd3*, and the fluorescence was observed. Scale bar, 5 μm. The white arrow with dotted lines indicated the areas used for linescan graph analysis. F) Appressorium collapse was analyzed in P131 and Δ*Mohrd3*. The conidia were induced on hydrophobic coverslips for 24 hpi, and the collapsed appressoria were assessed after exposure to 1, 2, and 3 M glycerol solution for 5 min. Error bars represent SD, n = 100. The significant differences were evaluated by two-tailed Student's *t*-test, ** $p < 0.01$; *** $p < 0.001$.

Figure S4

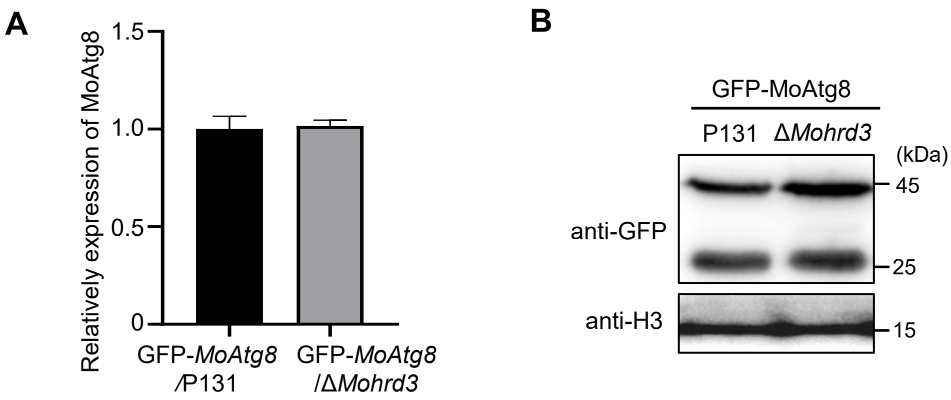

Figure S4. The relative expression level (A) and the protein accumulation (B) of MoAtg8 in the MoAtg8-GFP/P131 and MoAtg8-GFP/Δ*Mohrd3* strains.

Figure S5

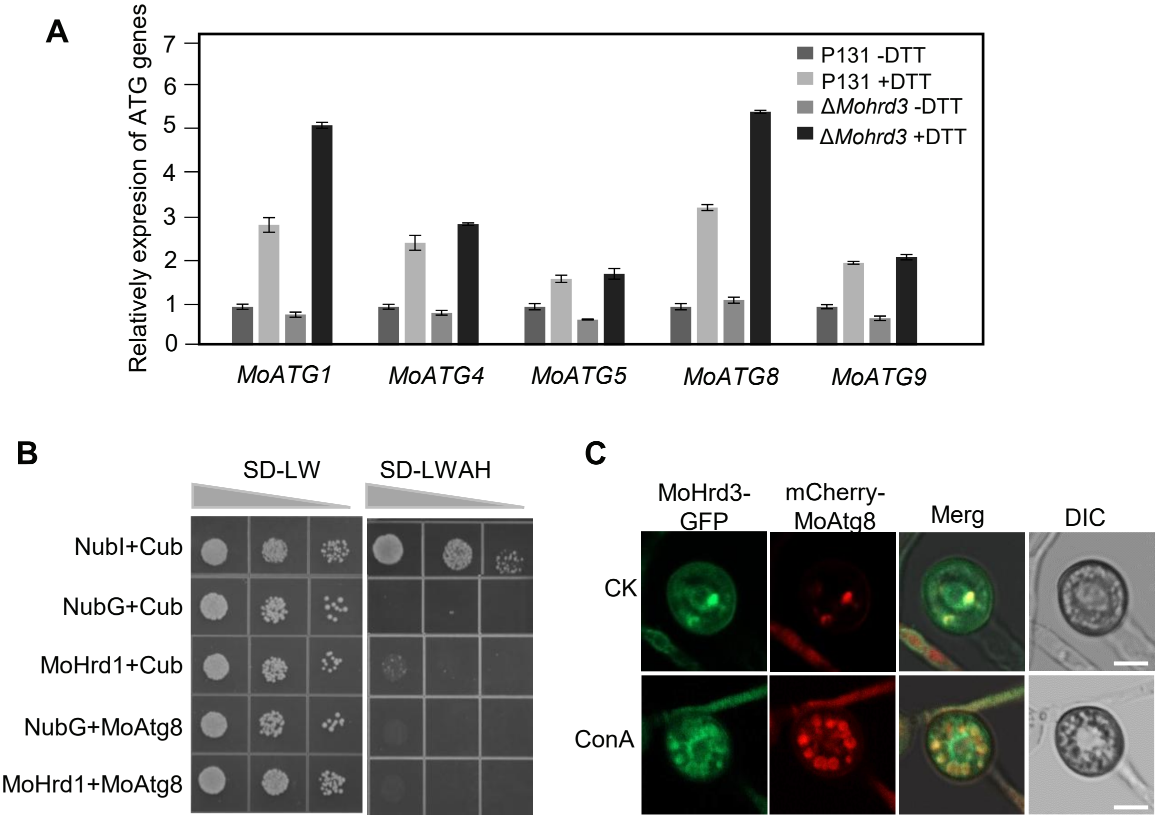

Figure S5. *MoHrd3* is required for autophagy. A) Several ATG genes are induced in Δ*Mohrd3* mutant. The relative expression levels of ATG genes were analyzed by qRT-PCR. Error bars represent SD. B) MoHrd1 didn't bind to MoAtg8 in yeast by split-ubiquitin yeast two-hybrid system. NubG/Cub co-transformation was used as negative controls. C) MoHrd3-GFP was co-localizated with mCherry-MoAtg8 in appressoria. Bar, 5 μm.

Figure S6

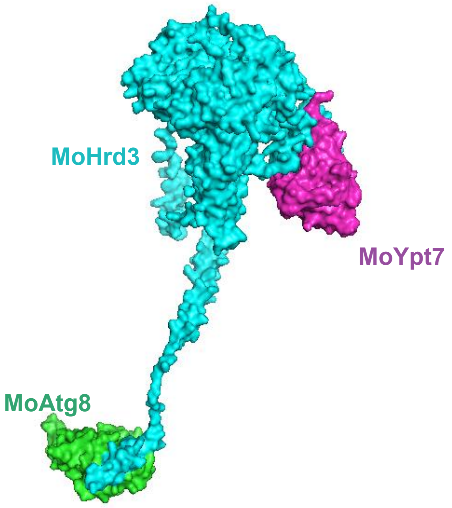

Figure S6. Molecular docking of MoHrd3, MoAtg8 and MoYpt7 complex. The blue molecular indicates MoAHrd3. The purple MoYpt7 protein and green MoAtg8 protein interact with N- or C-terminal of MoHrd3 respectively. Molecular docking was performed using ClusPro 2.0 (https://cluspro.org/signup.php), and the complex model was generated with PyMOL.

Figure S7

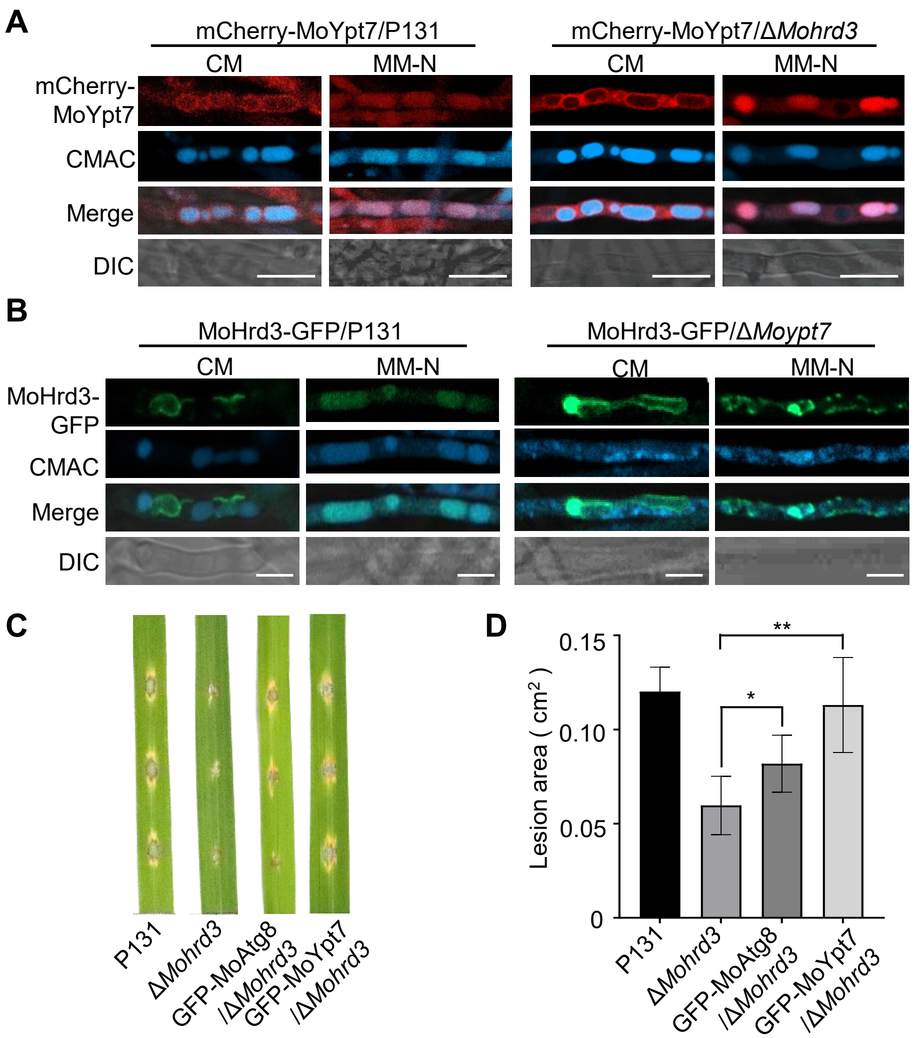

Figure S7. Overexpression of *MoAtg8* or *MoYpt7* partially restores the pathogenicity defect caused by Δ*Mohrd3*. A) The localization of mCherry-MoYpt7 in mycelia of P131 and Δ*Mohrd3*. mCherry-MoYpt7/P131 and mCherry-MoYpt7/Δ*Mohrd3* transformants were cultured in liquid CM for 36 h and then transferred to MM-N medium for 5 h before microscope observation. Mycelia were stained with CMAC to indicate the vacuoles. Scale bar, 10 μm. B) The localization of MoHrd3-GFP in mycelia of P131 and Δ*Mo*ypt7. MoHrd3-GFP/P131 and MoHrd3-GFP/Δ*Moypt7* transformants were cultured in liquid CM for 36 h and then transferred to MM-N medium for 5 h before microscope observation. Mycelia were stained with CMAC to indicate the vacuoles. Scale bar, 5 μm. C) Overexpression of *MoAtg8* or *MoYpt7* partially restores the reduced pathogenicity of Δ*Mohrd3*. Rice leaves were drop-inoculated with conidial suspensions (1×10^5 spores/ml) of the indicated strains and photographs were taken at 5 dpi. D) Statistical analysis of lesion area on rice leaves in (C). Error bars represent SD, n = 6. The significant differences were evaluated by two-tailed Student's *t*-test, $*p < 0.05$, $**p < 0.01$.

Figure S8

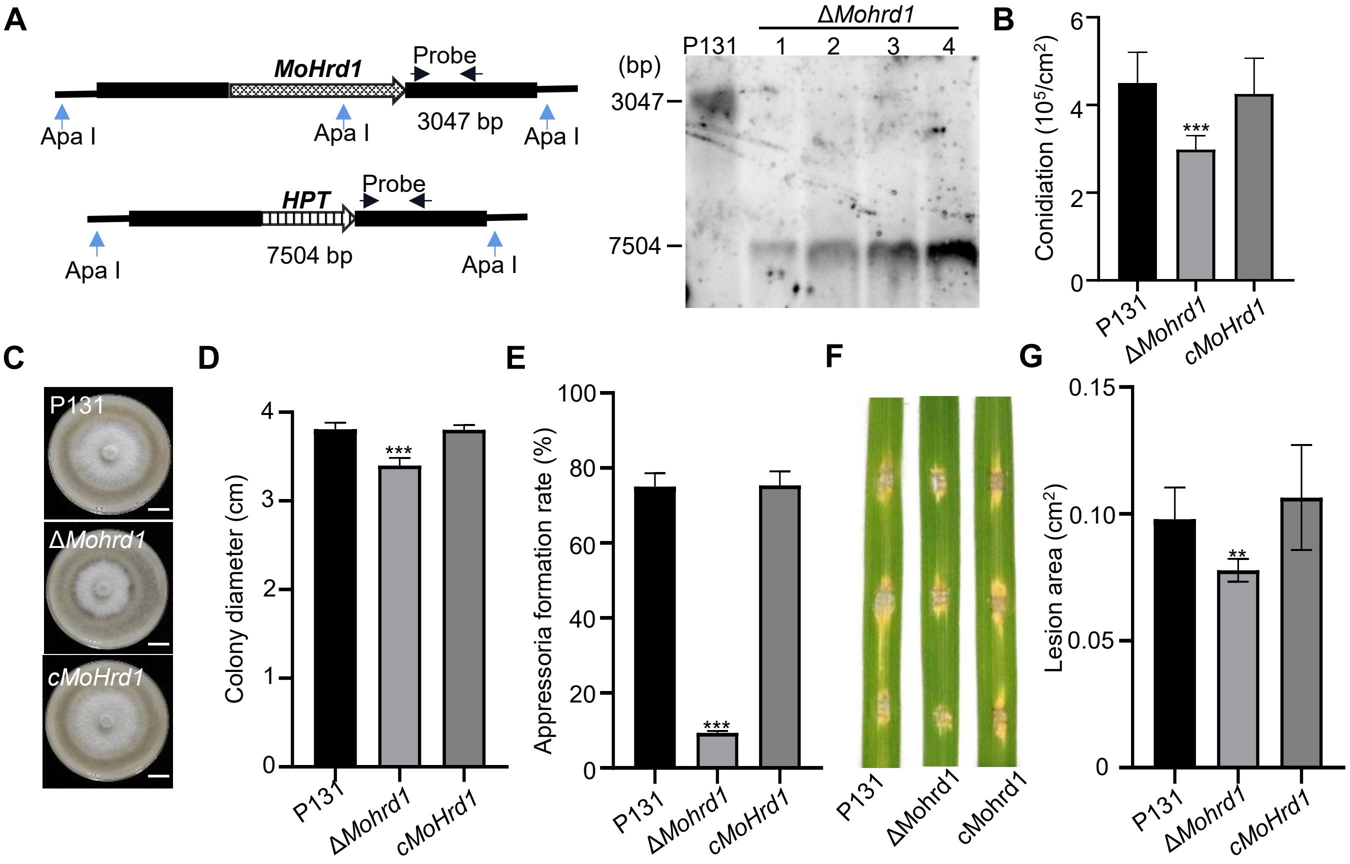

Figure S8. *MoHrd1* is important to growth, conidiation and pathogenicity in *M.oryzae*. A) Southern blot analysis of the *MoHrd1* deletion mutants. Apa I was used to digest the genomic DNA of P131 and *MoHrd1* knockout candidate transformants. The probe was amplified from DNA of P131. P131 and four *MoHrd1* knockout candidate transformants (number in 1, 2, 3, and 4) were used in this assay. B) Statistical analysis of conidia production of P131, Δ*Mohrd1* and *cMoHrd1*. Error bars represent SD, n = 6. The significant differences were evaluated by two-tailed Student's *t*-test, *** $p < 0.001$. C, D) Colony diameter was measured at 5 days on OTA plates. Scale bar, 1 cm. Error bars represent SD, n = 3. The significant differences were evaluated by two- tailed Student's *t*-test. *** $p < 0.001$. E) The appressorium formation rate of P131, Δ*Mohrd1* and *cMoHrd1*. Error bars represent SD, n = 300. The significant differences were evaluated by two-tailed Student's *t*-test, *** $p < 0.001$. F) Rice leaves were drop-inoculated with conidial suspensions (1×10^5 spores/ml) of the indicated strains and photographs were taken at 5 days post-inoculation (dpi). G) Statistical analysis of lesion area on rice leaves in (G). Error bars represent SD, n = 6. The significant differences were evaluated by two-tailed Student's *t*-test, ** $p < 0.01$.

Figure S9

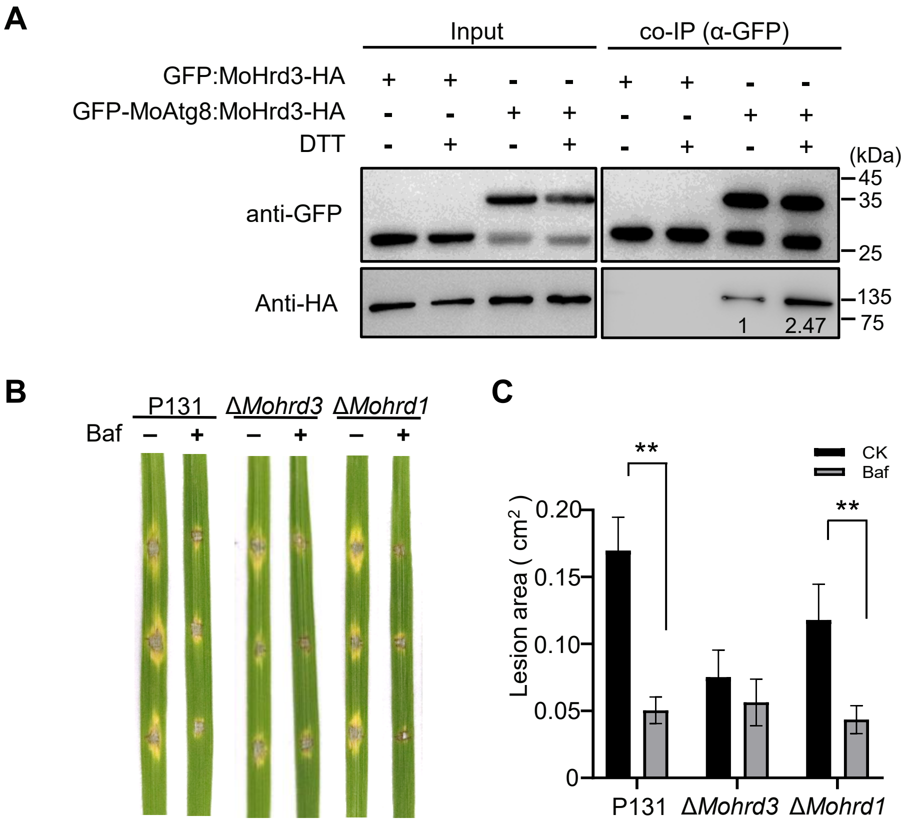

Figure S9. ER stress enhances the interaction between MoHrd3 and MoAtg8. A) DTT treatment enhanced the interaction between MoHrd3 and MoAtg8 in a co-IP assay. GFP: MoHrd3-HA and GFP-MoAtg8: MoHrd3-HA strains were treated with 2 mM DTT for 4 h in this assay. MoHrd3-HA was detected following GFP-MoAtg8 immunoprecipitation. The GFP: MoHrd3-HA strain was used as a negative control. B) Rice leaves were drop-inoculated with conidial suspensions (1×10^5 spores/ml) of the indicated strains with or without Baf, and photographs were taken at 5 dpi. C) Statistical analysis of lesion area on rice leaves in (B). Error bars represent SD, n = 6. The significant differences were evaluated by two-tailed Student's *t*-test, $**p < 0.01$.

Figure S10

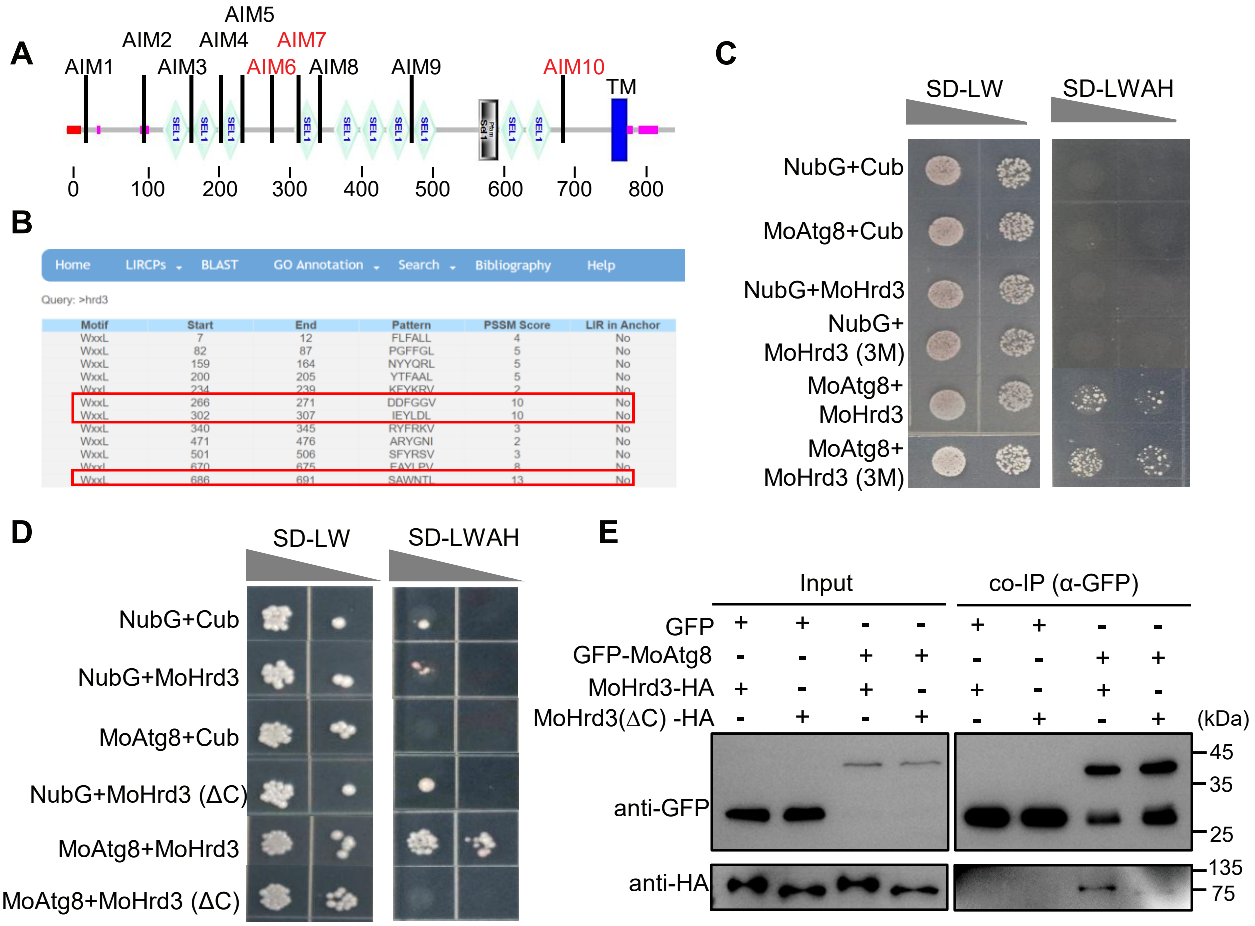

Figure S10. The C-terminal of MoHrd3 rather than AIM motifs is required for its interaction with MoAtg8. A) AIM motifs were indacated in protein schematic of MoHrd3. The MoHrd3 protein was predicted in SMART (http://smart.embl-heidelberg.de/). B) The AIM motifs of MoHrd3 were predicted in (https://ilir.warwick.ac.uk/search.php). The red boxes indicated three higher scored sites. C) The three high scored AIM motifs were not required for MoHrd3 and MoAtg8 interaction in yeast two-hybrid. MoHrd3 (3M), three high scored AIM mutant variant in which the AIM motif is mutated to alanine. D) The C-terminal domain was essential for MoHrd3 and MoAtg8 interaction in yeast two-hybrid. MoHrd3 (ΔC), a transformant strain with a deletion of the C-terminal 774-841 amino acid region of MoHrd3. E) The deletion of C-terminal impaired the interaction between MoHrd3 and MoAtg8 in a co-IP assay. GFP/Δ*Mohrd3*, GFP-MoAtg8/Δ*Mohrd3*, MoHrd3-HA/Δ*Mohrd3* and MoHrd3 (ΔC)-HA/Δ*Mohrd3* strains were used in this assay. The MoHrd3-HA and MoHrd3 (ΔC)-HA were detected following GFP-MoAtg8 immunoprecipitation.

Figure S11

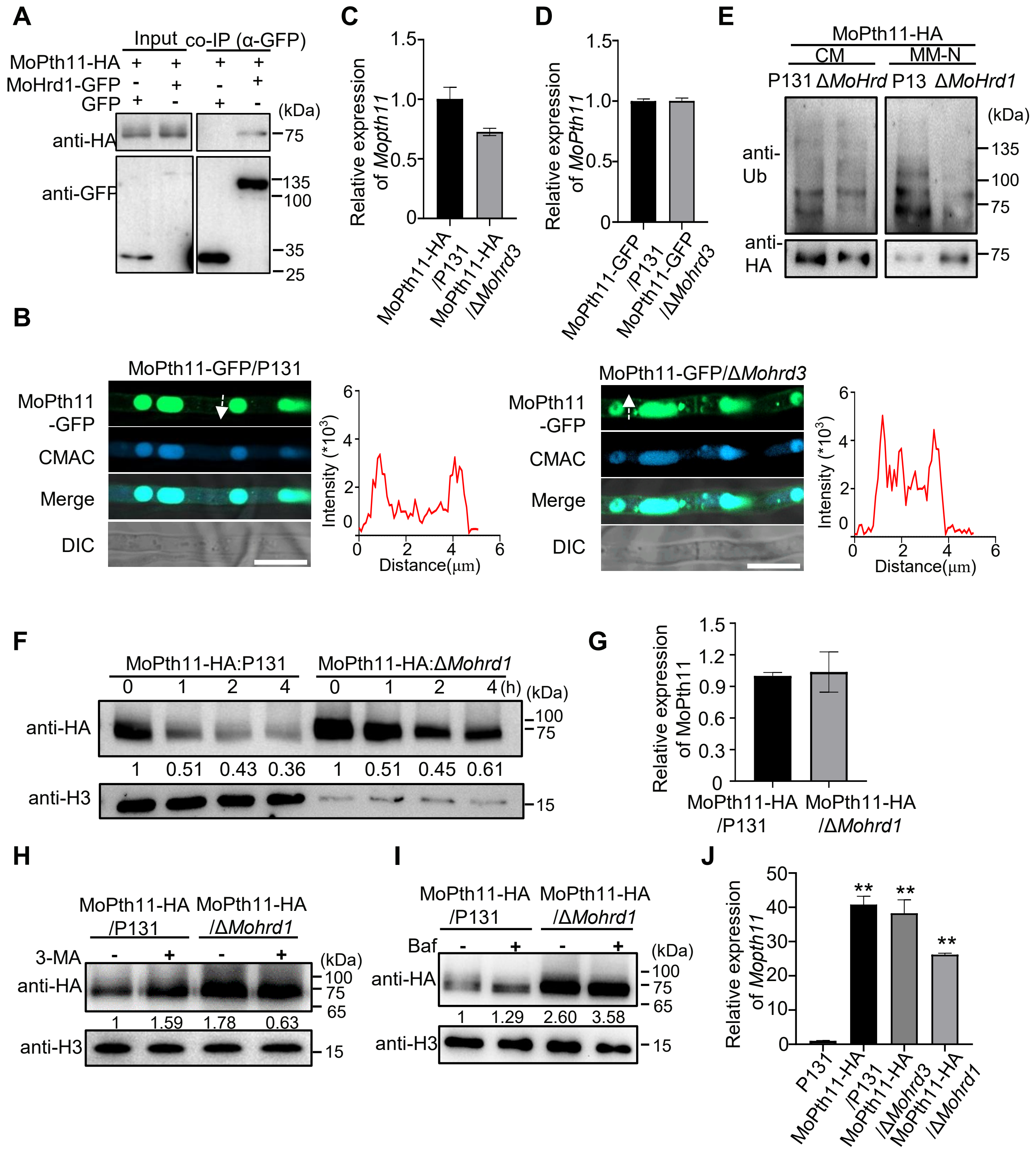

A
Input
co-IP (α-GFP)
MoPth11-HA
MoHrd1-GFP
GFP
(kDa)
anti-HA
anti-GFP
75
135
100
35
25
C
Relative expression of Mopth11
MoPth11-HA /P131
MoPth11-HA /ΔMohrd3
D
Relative expression of MoPth11
MoPth11-GFP /P131
MoPth11-GFP /ΔMohrd3
E
MoPth11-HA
CM
MM-N
P131 ΔMoHrd P13 ΔMoHrd1
anti-Ub
anti-HA
B
MoPth11-GFP/P131
MoPth11-GFP/ΔMohrd3
MoPth11 -GFP
CMAC
Merge
DIC
Intensity (*10^3)
Distance(μm)
F
MoPth11-HA:P131
MoPth11-HA:ΔMohrd1
0 1 2 4 0 1 2 4 (h)
anti-HA
1 0.51 0.43 0.36 1 0.51 0.45 0.61
anti-H3
15
G
Relative expression of MoPth11
MoPth11-HA /P131
MoPth11-HA /ΔMohrd1
H
MoPth11-HA /P131
MoPth11-HA /ΔMohrd1
3-MA
anti-HA
1 1.59 1.78 0.63
anti-H3
I
MoPth11-HA /P131
MoPth11-HA /ΔMohrd1
Baf
anti-HA
1 1.29 2.60 3.58
anti-H3
J
Relative expression of Mopth11
P131
MoPth11-HA /P131
MoPth11-HA /ΔMohrd3
MoPth11-HA /ΔMohrd1
**

Figure S11. MoHrd3 and MoHrd1 are required for the ubiquitination and degradation of MoPth11. A) MoHrd1 interacted with MoPth11 in a co-IP assay. MoPth11-HA/P131 and MoHrd1-GFP/ Δ*Mohrd1* strains were used in this assay. MoPth11-HA was detected following MoHrd1-GFP immunoprecipitation. (B) The localization of MoPth11-GFP in mycelia of P131 and Δ*Mohrd3*. MoPth11-GFP/P131 and MoPth11-GFP/Δ*Mohrd3* were cultured in liquid CM before microscope observation. Mycelia were stained with CMAC. All strains were treated with 0.1g/ml LatB for 1h. The white arrow with dotted lines indicated the areas used for linescan graph analysis. Bars =10 μm. C) The relative expression level of *MoPth11* in the MoPth11-HA/P131 and MoPth11-HA/Δ*Mohrd3* strains. D) The relative expression level of *MoPth11* in the MoPth11-GFP/P131 and MoPth11-GFP/Δ*Mohrd3* strains. E) The ubiquitination level of MoPth11 in the MoPth11-HA/P131 and MoPth11-HA/Δ*MoHrd1* was assayed using ubiquitin antibody. Total protein was subjected to HA-immunoprecipitation and then assessed using the indicated antibodies. All strains were cultured in liquid MM-N medium for 5 h. F) The protein level of MoPth11 in MoPth11-HA/P131 and MoPth11-HA/Δ*Mohrd1* strains. Strains were treated with 30 μM CHX for 0, 1, 2, 4 h. G) The relative expression level of *MoPth11* in the MoPth11-HA/P131 and MoPth11-HA/Δ*Mohrd1* strains. H) and I) MoHrd1 is required for the autophogic degradation of MoPth11. All strains were cultured in liquid CM medium with or without 500 μM 3-MA (H) or 300 nM Baf (I) for 4 h. Then the protein level of MoPth11 in these samples were detected. J) The relative expression level of *MoPth11* in the P131, MoPth11-HA/P131, MoPth11-HA/Δ*Mohrd3* and MoPth11-HA/Δ*Mohrd1* strains. The significant differences were evaluated by two-tailed Student's *t*-test. ** $p < 0.01$.
