# Supplementary material for: ERAD Component MoHrd3 Facilitates Pathogenicity and Establishes a Direct Regulation on Autophagy in Magnaporthe Oryzae
Source: Adv Sci (Weinh). 2026 Feb 23;13(24):e20627. doi: 10.1002/advs.202520627 (PMC13116320; doi:10.1002/advs.202520627)
Supplement: Supplementary file 2 — Supporting File 2: advs74457‐sup‐0002‐Table S1.docx. [file ADVS-13-e20627-s001.docx]

**Dataset EV1**

Candidate interaction proteins of MoHrd3 that identified through IP-MS.

| Gene ID | Description | Num. of matches |
| --- | --- | --- |
| MGG_06962 | GTP-binding protein ypt1 (203 aa) | 2 |
| MGG_07191 | GTP-binding protein ypt4 (291 aa) | 1 |
| MGG_01185 | GTP-binding protein ypt5 (222 aa) | 2 |
| MGG_08144 | Ras-like protein Rab7 (206 aa) | 1 |
| MGG_03694 | beclin-1 (504 aa) | 2 |
| MGG_03459 | Atg26p (1564 aa) | 1 |
| MGG_07916 | vacuolar protein sorting-associated protein 4 (428 aa) | 1 |
| MGG_06743 | vacuolar protein sorting-associated protein 5 (608 aa) | 3 |
| MGG_01434 | vacuolar protein sorting-associated protein 17 (611 aa) | 2 |
| MGG_09914 | vacuolar protein sorting-associated protein 33A (667 aa) | 1 |
| MGG_00839 | vacuolar protein sorting-associated protein 74(367 aa) | 1 |
| MGG_00905 | Sec14 cytosolic factor (344 aa) | 2 |
| MGG_02418 | vesicular-fusion protein SEC18 (836 aa) | 5 |
| MGG_06910 | protein transporter SEC23 (771 aa) | 3 |
| MGG_06569 | transporter sec24 (1035 aa) | 2 |
| MGG_04856 | protein transporter SEC61 subunit alpha (477 aa) | 7 |
| MGG_05320 | translocation protein sec63 (705 aa) | 2 |
| MGG_04004 | vacuolar protein 8 (560 aa) | 1 |
| MGG_04160 | vacuolar membrane-associated protein IML1 (1875 a) | 2 |
| MGG_03283 | vacuolar membrane protein (1009 aa) | 1 |
| MGG_12371 | vacuolar transporter chaperone 2 (816 aa) | 3 |
| MGG_00922 | vacuolar protease A (396 aa) | 4 |
| MGG_07536 | vacuolar aminopeptidase 1 (622 aa) | 1 |
