# Supplementary material for: ERAD Component MoHrd3 Facilitates Pathogenicity and Establishes a Direct Regulation on Autophagy in Magnaporthe Oryzae
Source: Adv Sci (Weinh). 2026 Feb 23;13(24):e20627. doi: 10.1002/advs.202520627 (PMC13116320; doi:10.1002/advs.202520627)
Supplement: Supplementary file 3 — Supporting File 3: advs74457‐sup‐0003‐Table S2.docx. [file ADVS-13-e20627-s002.docx]

| Dataset EV2  Oligonucleotide primers used in this study. | |
| --- | --- |
| Name | Sequence (5’- 3’) |
| hpt-up | GACAGACGTCGCGGTGAGTT |
| hpt-down | TCTGGACCGATGGCTGTGTAG |
| MoHrd3-LB-F | CTATTCTCTAGAACTAGTGGATCCGTTCAGGAACCCGGCGTG |
| MoHrd3-LB-R | CTTCAATATCAGTTGAATTCCTGCAGGACGAAATTGGTGTCGGGAC |
| MoHrd3-RB-F | ATGCCGACCGGGAACCAGTTAAGCTTGGGGTGTGATGCTTACACCAT |
| MoHrd3-RB-R | ACTAAAGGGAACAAAAGCTGGGTACCGTTTCTGGCATGGGGAGTG |
| MoHrd3-Check-F | TTGGTCAGCCTACGGTCT |
| MoHrd3-Check-R | CTGCCGGGAATTATGGATGC |
| MoHrd3-F | ATGGGCCGTGTCATTTTGT |
| MoHrd3-R | CTAATGACCAACTCCTCCA |
| MoHrd3-GFP-F | ACCGCCAAAGGATCCACTAGTATGGGCCGTGTCATTTTGT |
| MoHrd3-GFP-R | ACCAGCACCTCTAGAACTAGTATGACCAACTCCTCCAGCAAC |
| pEF1α-MoHrd3-GFP-F | CACCAAACCGCCAAAGGATCCATGGGCCGTGTCATTTTG |
| pEF1α-MoHrd3-GFP-R | TCTAGAACTAGTGGATCCATGACCAACTCCTCCAGC |
| MoHrd3-HA-F | CACCAAACCGCCAAAGGATCCATGGGCCGTGTCATTTTG |
| MoHrd3-HA-R | ACCTCTAGAACTAGTGGATCCATGACCAACTCCTCCAGC |
| Hrd3-nYFP-F | GGATCCACTAGTTCTAGAATGGGCCGTGTCATTTTG |
| Hrd3-nYFP-R | ACTTTTGCTCCATTCTAGAATGACCAACTCCTCCAGC |
| Hrd3-cYFP-F | GGATCCACTAGTTCTAGAATGGGCCGTGTCATTTTG |
| Hrd3-cYFP-R | ATCGTATGGGTACATTCTAGAATGACCAACTCCTCCAGC |
| pPR3N-MoHrd3-F | GTTCCAGATTACGCTGGATCCATGGGCCGTGTCATTTTGT |
| pPR3N-MoHrd3-R | AATTACATGACTCGAGGTCGACCTAATGACCAACTCCTCCAGC |
| MoHrd3-STE-F | ATGTAATGGCCATTACGGCCGGCCGTGTCATTTTGTTTCTT |
| MoHrd3-STE-R | TGCAGATGGCCGAGGCGGCCCCATGACCAACTCCTCCAGCAAC |
| MoHrd3-STE-3M-F1 | ATGTAATGGCCATTACGGCCGGCCGTGTCATTTTGTTTCTT |
| MoHrd3-STE-3M-R1 | GCCTTCACCGTATGCACCGCCAGCGTCGTCAGA |
| MoHrd3-STE-3M-F2 | TCTGACGACGCTGGCGGTGCATACGGTGAAGGC |
| MoHrd3-STE-3M-R2 | TTGTGGACCATCGCATCAAGAGCCTCAATGATGT |
| MoHrd3-STE-3M-F3 | ACATCATTGAGGCTCTTGATGCGATGGTCCACAA |
| MoHrd3-STE-3M-R3 | TCCGTTGGTGGCGGTATTCGCTGCACTCTTCG |
| MoHrd3-STE-3M-F4 | CGAAGAGTGCAGCGAATACCGCCACCAACGGA |
| MoHrd3-STE-3M-R4 | TGCAGATGGCCGAGGCGGCCCCATGACCAACTCCTCCAGCAAC |
| MoHrd3-STEΔC-F | ATGTAATGGCCATTACGGCCAGGCAGCACCGTCAACAACA |
| MoHrd3-STEΔC-R | TGCAGATGGCCGAGGCGGCCCCGTAGTAAACAAGAAATGCAA |
| pPR3N-MoHrd1-F | GTTCCAGATTACGCTGGATCCATGCGGTTAGCCTGGTATGC |
| pPR3N-MoHrd1-R | AATTACATGACTCGAGGTCGACCTAATCATCCTCATCCTCAACTTCTACA |
| MoHrd1-STE-F | ATGTAATGGCCATTACGGCCCGGTTAGCCTGGTATGCC |
| MoHrd1-STE-R | TGCAGATGGCCGAGGCGGCCCCATCATCCTCATCCTCAACTTC |
| MoHrd1-LB-F | CGCTCTAGAACTAGTGGATCCTATCTAGTGGTACGGTATCC |
| MoHrd1-LB-R | TCAATATCAGTTATCGAATTCACGCAGGTGCCTCACATCCC |
| MoHrd1-RB-F | CCAGTTATCAAGCTTATCGATTGATGACGACCTTTGATTATGTACTTT |
| MoHrd1-RB-R | AGGGAACAAAAGCTGGGTACCCGACGACGTTCCGAAAGTAGTG |
| MoHrd1-Check-F | AGGTTCGCCATCCATCAA |
| MoHrd1-Check-R | ATCCCACCTCAGAAGAGTCG |
| MoHrd1-F | CAACTTTACGAGAAGGCTTGGT |
| MoHrd1-R | ATGATTGTGGTCCGACTGC |
| MoAtg8-STE-F | ATGTAATGGCCATTACGGCCCGCTCCAAGTTCAAGGACGA |
| MoAtg8-STE-R | TGCAGATGGCCGAGGCGGCCCCCTCGACTTCCTCAAACAGGTCG |
| pPR3N-MoAtg8-F | AGAGTGGCCATTACGGCCCGGATGCGCTCCAAGTTCAAG |
| pPR3N-MoAtg8-R | GAGAGGCCGAGGCGGCCTCACTCGACTTCCTCAAA |
| mCherry-MoAtg8-F | ATGGACGAGCTGTACAAGATGCGCTCCAAGTTCAAG |
| mCherry-MoAtg8-R | GTAACGTTAAGTGCGGCCGCTCACTCGACTTCCTCAAA |
| nYFP-MoAtg8-F | GGATCCACTAGTTCTAGAATGCGCTCCAAGTTCAAG |
| nYFP-MoAtg8-R | ACTTTTGCTCCATTCTAGATCACTCGACTTCCTCAAA |
| pPR3N-MoYpt7-F | GTTCCAGATTACGCTGGATCCATGTCGTCCAGAAAGAAGGTT |
| pPR3N-MoYpt7-R | AATTACATGACTCGAGGTCGACTTAGCAGGCGCATCCATC |
| GFP-MoYpt7-F | CACCAAACCGCCAAAGGATCCATGTCGTCCAGAAAGAAGGTT |
| GFP-MoYpt7-R | TCTAGAACTAGTGGATCCGCAGGCGCATCCATCCCT |
| MoYpt7-HA-F | CACCAAACCGCCAAAGGATCCATGTCGTCCAGAAAGAAGGTT |
| MoYpt7-HA-R | ACCTCTAGAACTAGTGGATCCGCAGGCGCATCCATCCCT |
| nYFP-MoYpt7-F | GGATCCACTAGTTCTAGAATGTCGTCCAGAAAGAAGGTT |
| nYFP-MoYpt7-R | ACTTTTGCTCCATTCTAGATTAGCAGGCGCATCCATC |
| MoPth11-GFP-F | CACCAAACCGCCAAAGGATCCATGGTTGCATTCACCCGG |
| MoPth11-GFP-R | TCTAGAACTAGTGGATCCGATGAGACCACCGGGCAG |
| MoPth11-HA-F | CACCAAACCGCCAAAGGATCCATGGTTGCATTCACCCGG |
| MoPth11-HA-R | ACCTCTAGAACTAGTGGATCCGATGAGACCACCGGGCAG |
| MoPth11-RT-F | ACTTGCGTGCAGAAGAGTTG |
| MoPth11-RT-R | CACTGCGATTCTCAATGGGG |
| MoActin-RT-F | AGCCTTCAGTCCTGGGTC |
| MoActin-RT-R | TCGACGACGGTGCCA |
| MoKAR2-RT-F | AACGGTCTCGAGAACTATGC |
| MoKAR2-RT-R | TCCTTCTGCTCCTCAAAATC |
| MoPDI1-RT-F | GTTGTCGCTTACTTGTCCAG |
| MoPDI1-RT-R | CGTCAAATTTCTTGTCGAAG |
| MoSCJ1-RT-F | GGTCACGTAGAGATGGTCAA |
| MoSCJ1-RT-R | CCTGTATTTCTCCCACACTG |
| MoSIL1-RT-F | GACTTAGAGGGTCTGCCTGT |
| MoSIL1-RT-R | GAAGTACATGTCGTGGGAAA |
| MoATG1-RT-F | GGTCATGCGGAGATCAATGC |
| MoATG1-RT-R | TTGCTGCGTACGGAGGTAAG |
| MoATG4-RT-F | TCGGCGACTGAGACGACTTC |
| MoATG4-RT-R | TCCACGCATGCCAGTTCTCC |
| MoATG5-RT-F | CTTGTTCCCGAGCAGCAGAG |
| MoATG5-RT-R | AAGCCAGCCGTCTGGATAGG |
| MoATG8-RT-F | CCGCACTCATGTCGAGCATC |
| MoATG8-RT-R | AGGTGTTCTCGCCGGAGTAG |
| MoATG9-RT-F | GCTCGGCGATGAAGACATAG |
| MoATG9-RT-R | GGATTGGCTCCACTGTTCTC |
